# Multi‐omics‐based analysis of high grade serous ovarian cancer subtypes reveals distinct molecular processes linked to patient prognosis

**DOI:** 10.1002/2211-5463.13553

**Published:** 2023-02-24

**Authors:** Yuanshuo Alice Wang, Ryan Neff, Won‐min Song, Xianxiao Zhou, Sezen Vatansever, Martin J. Walsh, Shu‐Hsia Chen, Bin Zhang

**Affiliations:** ^1^ Department of Genetics and Genomic Sciences Icahn School of Medicine at Mount Sinai New York NY USA; ^2^ Mount Sinai Center for Transformative Disease Modeling Icahn School of Medicine at Mount Sinai New York NY USA; ^3^ Icahn Institute of Genomics and Multiscale Biology Icahn School of Medicine at Mount Sinai New York NY USA; ^4^ Department of Pharmacological Sciences Icahn School of Medicine at Mount Sinai New York NY USA; ^5^ The Mount Sinai Center for RNA Biology and Medicine Icahn School of Medicine at Mount Sinai New York NY USA; ^6^ Immunotherapy Research Center Houston Methodist Research Institute TX USA; ^7^ Cancer Center Houston Methodist Research Institute TX USA

**Keywords:** co‐expression network, drug repositioning, key regulator, molecular subtypes, ovarian cancer

## Abstract

Despite advancements in treatment, high‐grade serous ovarian cancer (HGSOC) is still characterized by poor patient outcomes. To understand the molecular heterogeneity of this disease, which underlies the challenge in selecting optimal treatments for HGSOC patients, we have integrated genomic, transcriptomic, and epigenetic information to identify seven new HGSOC subtypes using a multiscale clustering method. These subtypes not only have significantly distinct overall survival, but also exhibit unique patterns of gene expression, microRNA expression, DNA methylation, and copy number alterations. As determined by our analysis, patients with similar clinical outcomes have distinct profiles of activated or repressed cellular processes, including cell cycle, epithelial‐to‐mesenchymal transition, immune activation, interferon response, and cilium organization. Furthermore, we performed a multiscale gene co‐expression network analysis to identify subtype‐specific key regulators and predicted optimal targeted therapies based on subtype‐specific gene expression. In summary, this study provides new insights into the cellular heterogeneity of the HGSOC genomic, epigenetic, and transcriptomic landscapes and provides a basis for future studies into precision medicine for HGSOC patients.

## Graphical abstract

We integrated multiomics information to identify seven novel HGSOC molecular subtypes using a multiscale clustering method. These subtypes have distinct survival, genetic, and epigenetic patterns. We also constructed a gene co‐expression network to identify subtype‐specific key regulators. We further predicted and validated targeted therapies for HGSOC molecular subtypes. This study provides a foundation for pecision medicine in HGSOC.

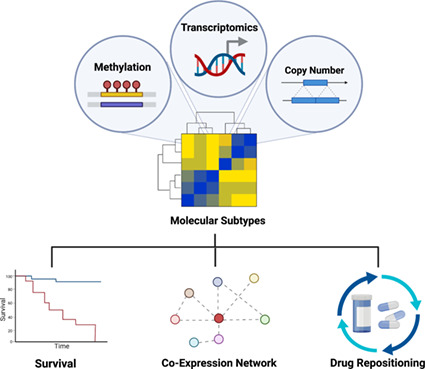

**Abbreviations**
ARI: adjusted Rand Index
BH: Benjamini–Hochberg
CAF: cancer‐associated fibroblast
CNA: copy number alteration
CTL: cytotoxic T‐lymphocyte
D2R: dopamine D2 receptor
DEG: differentially expressed gene
DMP: differential methylated probe
ECM: extracellular matrix
EMT: epithelial‐to‐mesenchymal transition
EMUDRA: Ensemble of Multiple Drug Repositioning Approaches
FC: fold change
FDR: false discovery rate
FET: Fisher's exact test
GDC: Genomic Data Commons
HGSOC: high‐grade serous ovarian cancer
Hh: hedgehog
HRD: homologous recombination deficiency
KDA: key driver analysis
KW: Kruskal–Wallis
MDSC: myeloid‐derived suppressor cell
megena
: multiscale embedded gene co‐expression network analysis
MHC: major histocompatibility complex
MSigDB: Molecular Signatures Database
MWINA: Multiscale Weighted Interaction Network Analysis
NSCLC: non‐small‐cell lung cancer
OS: overall survival
PARPi: poly‐ADP ribose polymerase inhibitors
PFNs: Planar Filtered Networks
RB: Reichardt‐Bornholdt
SPD: shortest path distance
TCGA: The Cancer Genome Atlas
TGF‐beta: transforming growth factor‐beta

Ovarian cancer is the most lethal gynecological cancer in the United States, with over 14 000 estimated deaths in 2018 that make up 5% of total cancer‐related deaths in women [1]. Though the 5‐year survival rate for localized disease is over 90%, the vast majority of patients are not diagnosed until the tumor has already progressed to distant stage disease, for which the average 5‐year survival drops to below 30% [1]. High‐grade serous ovarian cancer (HGSOC) accounts for the majority of mortalities from ovarian cancer, and its overall survival has shown little improvement over the past several decades. Despite initial effective treatment with platinum‐based chemotherapy, resistance arises in a large majority (80–90%) of patients with late‐stage disease [1]. Although poly‐ADP ribose polymerase inhibitors (PARPi) have been a recent treatment of HGSOC carrying homologous recombination deficiency (HRD), therapeutic options approaches for remaining HGSOC are still limited [1, 2]. Therefore, multiomics approaches are expected to predict disease processes for enabling precision‐guided therapeutic strategies.

Recently, there have been efforts directed at identifying molecular subtypes of HGSOC that may further identify and predict unanticipated susceptibilities. Tothill et al. were one of the first groups to stratify high‐grade tumors into subtypes with distinct molecular signatures, followed by The Cancer Genome Atlas (TCGA) Research Network, which also identified a set of HGSOC subtypes with similar molecular signatures from both the TCGA and Tothill cohorts [3, 4, 5]. However, the subtypes based on the TCGA transcriptomic data did not differ significantly in survival, though their miRNA‐based subtypes did [5]. Later, studies focusing on transcriptomic and/or multiomic data identified subtypes with molecular characteristics similar to those by Tothill et al and The Cancer Genome Atlas Research Network [6, 7, 8, 9, 10, 11, 12, 13]. Though previous studies successfully identified a poor surviving subtype enriched for epithelial‐to‐mesenchymal transition (EMT), extracellular matrix (ECM), and transforming growth factor‐beta (TGF‐beta)‐related genes, our clustering method allows for more comprehensive molecular characterization to compare and contrast all the identified subtypes and to expand the range of innovative subtype‐specific treatment strategies [7, 12, 14, 15, 16, 17, 18, 19]. Moreover, there remains opportunities to identify additional subtypes of HGSOC [12, 18].

Using a novel clustering approach, Multiscale Weighted Interaction Network Analysis (MWINA) [20], we developed a rigorous systems biology framework for analyzing multiomics data from HGSOC to reveal seven HGSOC patient subtypes that are significantly distinct in their overall survival (OS). Compared with the previously published HGSOC subtypes, the new subtypes show unique combinations of activated or repressed biological processes such as cell cycle, epithelial‐to‐mesenchymal transition, immune activation, interferon response, and cilium organization. We then used subtype‐specific gene expression to identify molecular signatures, networks, key regulators, and therapeutic compounds for each subtype and showed that even subtypes with similarly poor or favorable clinical outcomes are characterized by different molecular pathways. Finally, we utilized the subtype‐specific gene expression profiles to predict drugs against each subtype. This study provides not only new insights into the processes underlying the molecular heterogeneity of HGSOC but also identifies potential therapeutics for HGSOC subtypes, representing a significant advance toward precision medicine for ovarian cancer.

## Results

### Identification of HGSOC molecular subtypes

For sample clustering, we used survival‐associated features from the mRNA expression, microRNA expression, copy number alteration (CNA), and DNA methylation data from 512 TCGA HGSOC samples (TCGA‐HGSOC) selected by Zhang et al. [14]. The prognostic significance of each individual feature was assessed by univariate Cox proportional hazard model analysis (*P* < 0.05) relative to patient survival [14]. A total of 4526 features were used, including the expression of 1651 mRNA genes, 140 microRNAs, 2191 somatic CNAs, and 455 DNA methylation sites. Feature values were then normalized with respect to the expression of normal controls as described previously [14].

**Fig. 1:**
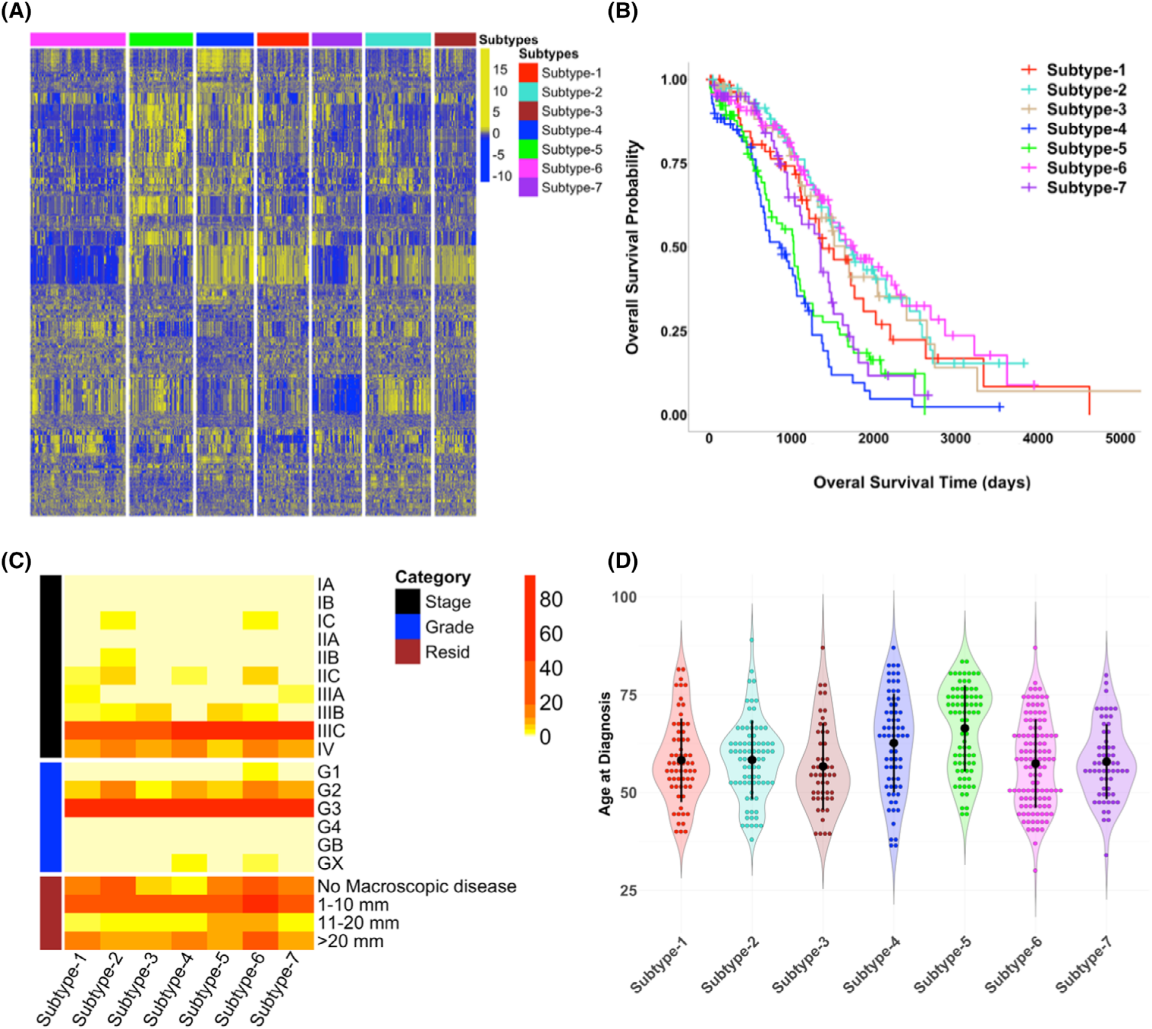
MWINA‐based HGSOC subtypes with distinct molecular profiling patterns and overall survival (OS), but similar clinical traits. (A) Multiscale weighted clustering of 512 TCGA ovarian cancer patients based on previously published features significantly associated with survival. These include mRNA and miRNA expression, copy number segments, and DNA methylation positions. Columns represent samples organized by subtype membership, and rows represent *z*‐score normalized feature expression. (B) Kaplan–Meier survival curves showing OS probabilities for seven novel ovarian cancer subtypes. Censored data are represented by “+” to indicate time to last follow‐up. Chi‐squared *P*‐value < 4.21 × 10^−11^. (C) Clinical traits for each ovarian cancer subtype. Clinical traits shown include tumor stage, tumor histological grade, and residual tumor size. Color gradient corresponds to number of patients. (D) Distribution for age at diagnosis for patients categorized by molecular subtype, with mean age ± one standard deviation shown for each subtype.

A new clustering approach, Multiscale Weighted Interaction Network Analysis (MWINA), was utilized to identify novel HGSOC subtypes from the TCGA‐HGSOC cohort. Briefly, MWINA optimizes for network modularity, *Q*(γ_RB_), and Reichardt‐Bornholdt parameter, γ_RB_, controls the resolution of the optimal solution [21] (see Methods for details). By exploiting γ_RB_ across a range of 0.1–4, 60 sets of HGSOC patient subtypes with varying degrees of compactness were generated. These sets were then ranked based on the significance of the subtype OS (defined as the time between initial surgical resection to date of death or date of last follow‐up) as demonstrated by the survival chi‐squared statistic. Finally, we selected the highest ranked set that contains seven subtypes with highly varied molecular signatures and significantly distinct OS (chi‐squared *P* < 4.21 × 10^−11^). (Fig. 1A,B, Table 1).

**Table 1:** Summary of clinical, transcriptomic, and epigenetic traits for all subtypes.

Subtype	# Samples	Mean age at diagnosis	Mean OS (days)	miRNA dysreg.^a^	Methyl. dysreg.^a^	Immune response	ECM	Cell cycle	Cilium
1	62	58.26	1067.05	Low	Low	Low	–	High	Med‐High
2	79	58.32	1284.11	Moderate	Moderate	High	Low	–	–
3	50	56.68	1177.28	Low	Low	–	–	–	–
4	69	62.65	764.64	High	High	High	High	Low	Low‐Med
5	77	66.42	841.1	High	High	Low	Low	High	–
6	115	57.39	1164.54	Low	Moderate	–	Low	Low	High
7	60	57.88	946.68	Low	Moderate	–	–	–	–

^a^ The level of miRNA or methylation dysregulation is determined by the number of differentially expressed miRNAs or methylation sites for each subtype (Table S4), such as subtypes with high, intermediate, and low numbers of DE‐miRNAs and DMPs are designated as having high, moderate, and low dysregulation. The immune response, ECM, cell cycle, and cilium categorizations are defined by the number of upregulated subtype‐specific DEGs that are enriched for these categories (Fig. 2A). Subtypes with downregulated DEGs that are enriched for these categories are labeled as “low” (Fig. 2B).

The stability of the MWINA‐based subtypes was assessed by subsampling. MWINA was performed on each of 50 datasets generated by randomly subsampling for 80% of all features and 80% of all patients. A concordance matrix was then generated across all the bootstrapped clusters to represent the probabilities that any pair of two patients are consistently found in the same cluster (Fig. S3A). From the bootstrapping outputs, empirical *P*‐values were computed to evaluate cluster concordances across the 50 datasets. All the MWINA‐based subtypes yielded *P*‐values < 10^−6^, demonstrating the robustness of the identified subtypes.

To check whether the subtypes were biased toward clinical traits, we tested whether the seven subtypes can be distinguished by tumor stage, tumor histological grade, residual tumor size, or age at diagnosis. Kruskal–Wallis (KW) test shows that there is no significant difference in tumor stage, histological grade, or residual tumor size among the subtypes (Fig. 1C; Table S1), but age at diagnosis is significantly different among the subtypes and affects OS (Cox proportional hazard *P* = 0.0020, HR = 1.02), with Subtype‐4 and Subtype‐5 having the highest mean age at diagnosis (Fig. 1D, Table 1). However, removing Subtype‐4 and Subtype‐5 eliminated this covariate effect (Cox proportional hazard *P* = 0.34, HR = 1.01, KW‐test *P* = 0.8124) among the rest of the five subtypes (Table S1). Furthermore, the age at diagnosis for each pair of subtypes was significantly different (Student's *t*‐test *P* < 0.05) only for comparisons with Subtype‐4 or Subtype‐5 but not for all other subtype pairs (Fig. S3B), suggesting that age at diagnosis is not a significant predictor of subtype membership for the other five subtypes. Lastly, Cox proportional hazard analysis showed a significant effect of residual disease on OS in the overall cohort (*P* = 0.0042, HR = 1.19), but not within subtypes 2, 4, 5, and 6 (*P* = 0.089, HR = 1.14). Although subtype‐6 has fewer samples with residual disease compared with the others, this result suggests that membership within the subtypes with the shortest and longest OS may not be due to amount of residual tumor alone.

In comparison with the published TCGA‐HGSOC subtypes by Zhang et al. [14], redistribution of the TCGA‐HGSOC samples by the MWINA cluster algorithm improved the subtype prognostic significance by several orders of magnitude (MWINA chi‐squared *P* < 4.21 × 10^−11^ vs. Zhang et al. 2013 chi‐squared *P* = 2.96 × 10^−7^) (Fig. S3C). In addition, the MWINA‐based subtypes with poor OS do not have significantly more late‐stage disease patients compared with other MWINA‐based subtypes (Fig. 1C; Table S1), which is in contrast to the poor prognosis subtype identified by Zhang et al. [14] that contains more patients in stages IIIC and IV. Lastly, we identified subtypes with significant variations in immune cell activation, which was not a focus of the subtypes from Zhang et al. [14]. This shows that the MWINA‐based subtypes are not duplication of the subtypes from Zhang et al. [14], but rather a redistribution of these patients to form even more prognostically distinct groups.

### Molecular signatures of the HGSOC subtypes

**Fig. 2:**
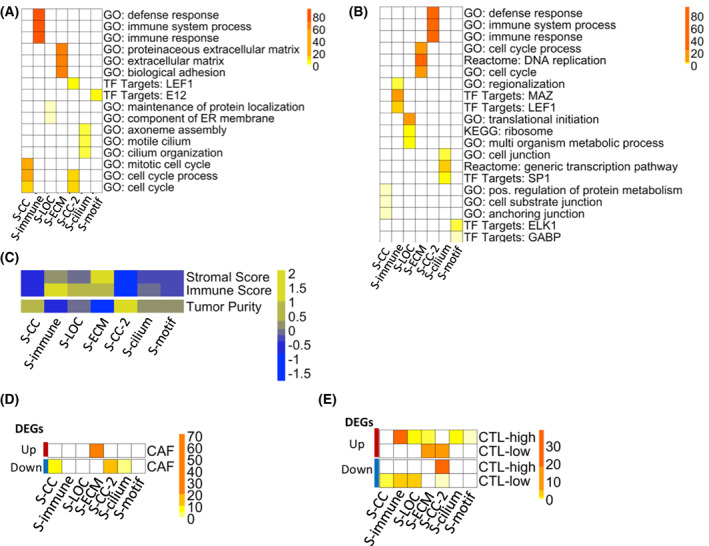
HGSOC subtypes have distinct gene expression signatures and cell type compositions. (A, B) Enrichment of MSigDB gene sets in the (A) upregulated and (B) downregulated DEGs for each subtype. DEGs were identified by comparing the samples in each subtype with the rest of the samples (BH‐corrected *P* < 0.05, fold change ≥ 1.2) and enrichment is determined via Fisher's exact test (FET) for overlapping genes. Color gradient shows −log_10_(FET *P*‐value). (C) Heatmap showing the mean estimate Stromal, Immune, and Tumor Purity scores for each subtype. All scores are *z*‐score normalized across subtypes. (D, E) Heatmaps showing enrichment of subtype‐specific up‐ (red, top) and down‐ (blue, bottom) regulated DEGs for (D) Cancer‐associated fibroblast (CAF) markers under regulation by TGF‐beta, and for genes characteristic of (E) cytotoxic T‐lymphocyte (CTL) high and low HGSOC tumors. Enrichment is determined via FET. Color gradient shows −log_10_(FET Bonferroni‐corrected *P*‐value). See also Fig. S4.

To understand the underlying molecular processes of each subtype, we performed differential gene expression (DEG) analysis on the transcriptomic data. We compared the samples within each subtype with those from the rest of the six subtypes in the full RNA‐Seq data of the 420 samples from the TCGA‐HGSOC cohort and also performed DEG analysis on the microarray data between the samples in each subtype against the normal controls (*n* = 8). The Molecular Signatures Database (MSigDB) was used to identify pathways and functions uniquely enriched in differentially expressed genes per subtype (BH‐corrected *P* < 0.05 and fold change ≥ 1.2) (Fig. 2A,B). The samples in Subtype‐4 are enriched for extracellular matrix (ECM) functions including adhesion, vasculature development and cell movement compared with the rest of the subtypes and normal samples (Fig. 2A; Fig. S4A). Upregulation of these processes likely contributes to the poor prognosis of the patients in this subtype, as many of these genes have been implicated in epithelial‐to‐mesenchymal transition (EMT) and metastasis [22]. By contrast, though the patients in Subtype‐5 also have poor OS (Fig. 1B, Table 1), they are characterized by upregulated cell cycle process genes and downregulated genes in defense response and immune system process (Fig. 2A,B; Fig. S4B). Similar to Subtype‐5, Subype‐1 also has upregulated cell cycle genes but it has downregulated genes involved in cell substrate and anchoring junctions (Fig. 2A,B). Subtype‐2, Subtype‐3, and Subtype‐6, with favorable survival (Fig. 1B, Table 1), have upregulated immune activation, protein localization, and cilium organization, respectively (Fig. 2A). Finally, a significant number of genes upregulated in Subtype‐7 have the conserved CAGGTG motif, and many of the downregulated genes have the conserved SCGGAAGY motif corresponding to ELK1 transcription factor binding site (Fig. 2A,B). Due to their distinct molecular pathways, Subtypes‐1, 2, 3, 4, 5, 6, and 7 are renamed as S‐CC (cell cycle), S‐immune, S‐LOC (localization), S‐ECM, S‐CC‐2, S‐cilium, and S‐motif, respectively. The results show that subtypes with similar prognoses are characterized by distinct molecular pathways (Fig. S4C–F).

As previously shown, HGSOC tumors can have a high amount of stromal and immune cell infiltration from the tumor microenvironment [23]. To explore whether subtype‐specific gene expression signatures are due to contributions from the tumor microenvironment, we examined cell type composition of each subtype. Using the estimate R package [24], we computed stromal scores, immune scores, and tumor purity scores as proxies for tumor composition (Fig. 2C). Although subtype tumor purity scores were not directly correlated with survival outcome (Pearson correlation rho = 0.045, *P*‐value = 0.33), the KW‐test shows that the subtype stromal, immune, and tumor purity scores are significantly different among the seven subtypes (Table S1).

The subtype‐specific immune and ECM gene signatures are likely due to immune and stromal cell infiltrates from the tumor microenvironment. The upregulated DEGs from S‐ECM significantly overlap (Fisher's exact test (FET) Bonferroni‐corrected *P* = 1.01 × 10^−72^) with CAF‐specific genes that are under TGF‐beta regulation [25] (Fig. 2D). By contrast, there is no overlap between the TGF‐beta regulated CAF‐specific genes and DEGs from other subtypes (Fig. 2D). The DEGs from S‐immune significantly overlap (FET Bonferroni‐corrected *P* < 0.05) with the differentially expressed genes in ovarian tumors rich in cytotoxic T‐lymphocytes (CTLs) [26], demonstrating the presence of an infiltrating CTL population in the S‐immune subtype (Fig. 2E). The DEGs from other subtypes with similar OS as S‐immune, such as S‐LOC and the S‐cilium, also overlap with markers corresponding CTL‐high and CTL‐low states (Fig. 2E). By contrast, the upregulated DEGs in S‐ECM have significant overlap with the CTL‐low gene signature, suggesting that the immune cell signature in S‐ECM does not originate from an infiltrating CTL population (Fig. 2E). Instead, the immune signature in the S‐ECM is likely from a myeloid‐derived suppressor cell (MDSC) population, as demonstrated by the significant overlap (FET Bonferroni‐corrected *P* < 2.06 × 10^−14^) of the S‐ECM upregulated DEGs with genes overexpressed in MDSCs from non‐small‐cell lung cancer (NSCLC) (data accessible at NCBI GEO database, accession GSE79404) (Fig. S4G) [27, 28]. Lastly, the S‐CC‐2 seems to lack both infiltrating CTLs and MDSCs, as shown by the low estimate stromal and immune scores, as well as downregulation of CAF, CTL, and MDSC‐specific genes in its DEG signature (Fig. 2D,E; Fig. S4G,H).

### Subtype‐specific gene networks and key regulators

**Fig. 3:**
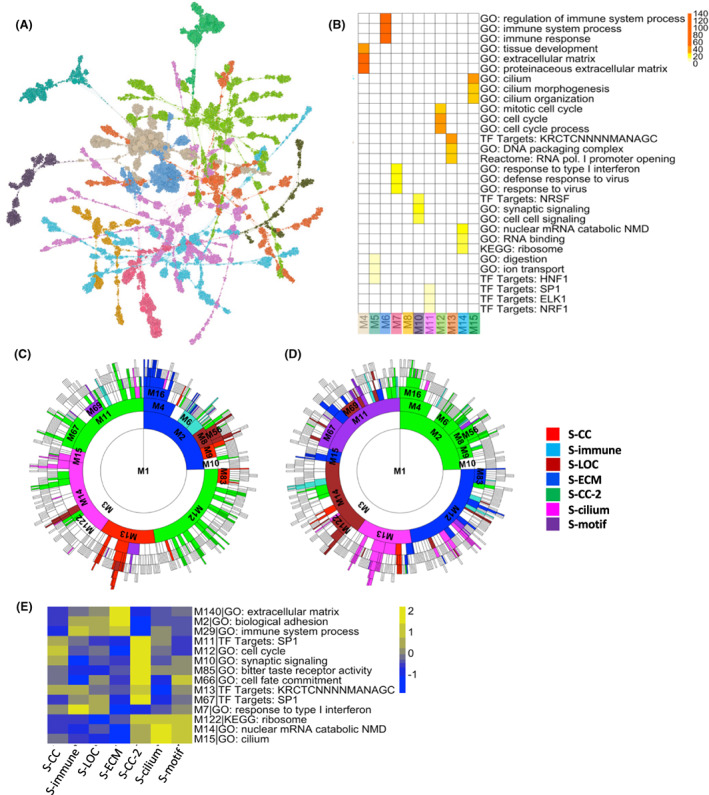
Characterization of the HGSOC subtype gene signatures of by the megena co‐expression network. (A) Graphical representation of the global megena gene network based on TCGA HGSOC mRNA expression. Node colors represent gene module membership, and node size is proportional to node degree. (B) Heatmap showing enrichment of module genes for MSigDB gene sets. Modules correspond to those shown in Fig. 3A. Module enrichment is determined via Fisher's exact test (FET) for overlapping genes. Color gradient shows −log_10_(FET Bonferroni‐corrected *P*‐value). (C, D) Sunburst plots showing overlap of subtype‐specific (C) upregulated or (D) downregulated DEGs with megena network module genes. Colors correspond to subtypes whose DEGs are most enriched for that module. Enrichment is ranked by FET *P*‐value. Modules with FET Bonferroni‐corrected *P*‐value > 0.05 are not colored. (E) Subtype mean expression of hub genes from select megena gene network modules. Mean expression is normalized by *z*‐score across subtypes. See also Figs S5 and S6.

To uncover gene–gene coexpression/co‐regulation relationships in HGSOC, multiscale embedded gene co‐expression network analysis (megena) was performed to construct a global gene–gene correlation network from the transcriptomic data of all patients [29] (Fig. 3A, Table 2). While the RNA‐Seq data were available for only 420 TCGA HGSOC samples for building the megena network, this subpopulation and the 512 patients used for subtype clustering are sufficiently similar to make the network an accurate clinical representation of the entire TCGA‐HGSOC cohort (Table S2). megena identified 826 gene modules, which were significantly enriched for a variety of functions such as ECM (module M4), immune system process (module M6), interferon response (module M7), cell cycle (module M12), and cilium organization (module M15) (Fig. 3B).

**Table 2:** Key resources.

Reagent or resource	Source	Identifier
Software and algorithms		
Deposited data	Zhang et al. [14]	https://doi.org/10.17632/67yzwc826b.2
megena	Song et al. [29]	http://research.mssm.edu/multiscalenetwork/packages/MEGENA_1.1.tar.gz
estimate	Yoshihara et al. [24]	https://bioinformatics.mdanderson.org/estimate/
MSigDB	Broad Institute	http://software.broadinstitute.org/gsea/msigdb
limma	Ritchie et al. [74]	https://bioconductor.org/packages/release/bioc/html/limma.html
lumi	Du et al. [73]	https://bioconductor.org/packages/release/bioc/html/lumi.html
gistic2.0	Mermel et al. [36]	GISTIC2.0
genepattern notebook	Reich et al. [78]	http://genepattern‐notebook.org/
emudra	Zhou et al. [37]	https://doi.org/10.7303/syn11510888
clue repurposing	CLUE	https://clue.io/repurposing‐app

The co‐expressed gene modules significantly overlap with many DEG signatures of the subtypes. As expected, the signatures of these subtypes with similar OS are enriched for different sets of gene modules. S‐ECM and S‐CC‐2, the two subtypes with the poorest prognosis, have opposite patterns of DEG enrichment of the ECM modules (M4, M16, and M140) and cell cycle module (M12 and M12‐child modules) genes (Fig. 3C,D; Figs S5 and S6). For the subtypes with good prognosis, S‐immune DEG signature shows upregulation of the immune system (M6) and interferon response (M7) modules, in contrast to S‐cilium, which shows upregulation of ribosome and translation (M14) and cilium (M15) module genes and downregulation of DNA packaging and transcription (M13) genes (Fig. 3C,D; Figs S5 and S6). For subtypes with intermediate OS, S‐CC demonstrates upregulation in cell cycle (M83) as well as DNA packaging and transcription (M13, M114) module genes, S‐LOC shows downregulation in RNA processing (M69) and ribosome and protein translation (M14, M122, and M421) module genes, and S‐motif shows upregulation in targets of transcription factor SP1 (M11 and M67) modules (Fig. 3C,D; Fig. S5). Trends in the mean expression of megena module hub genes for each subtype also closely match the enriched modules, showing that these globally identified module hub genes are likely also subtype key hubs (Fig. 3E).

**Fig. 4:**
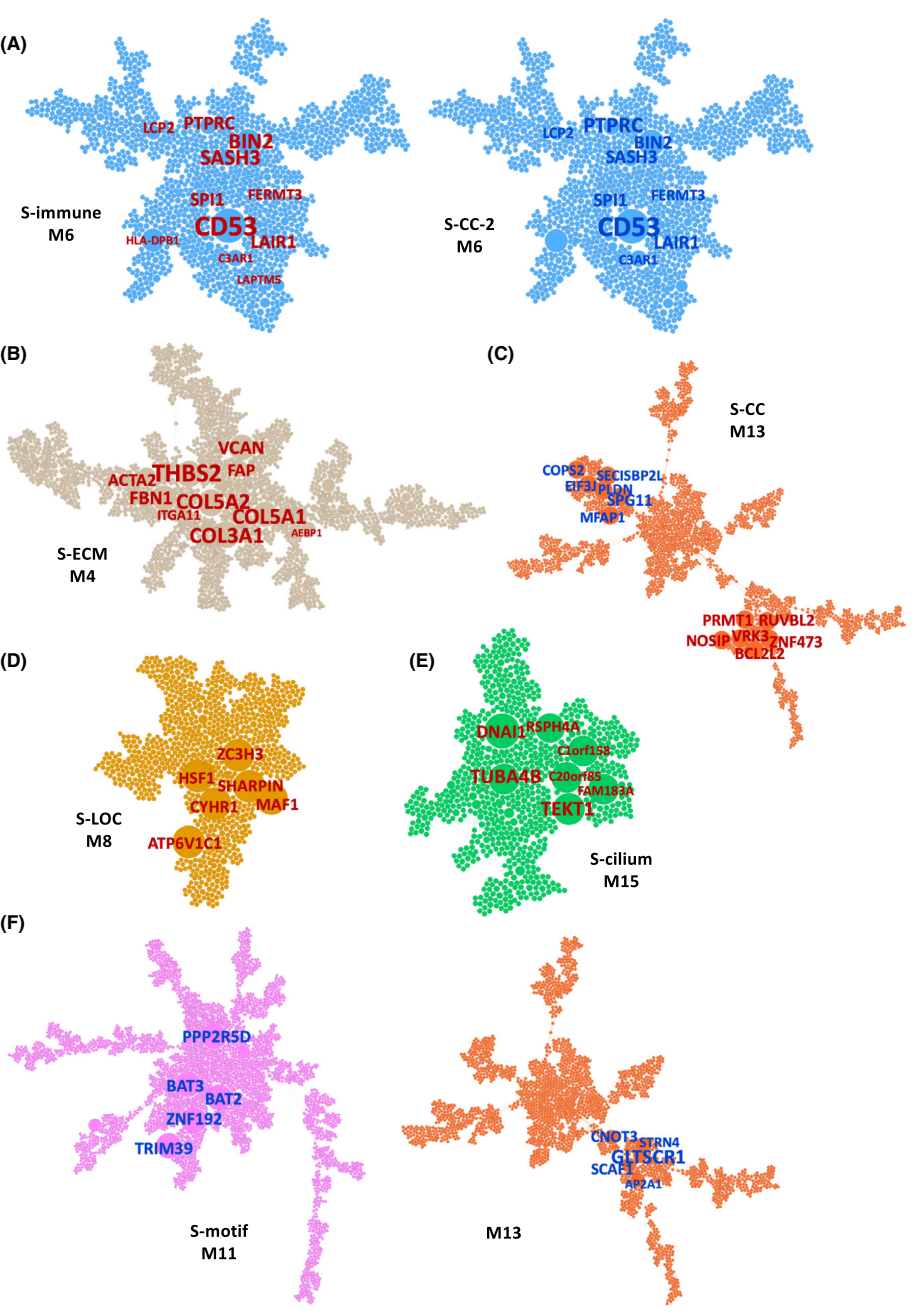
Predicted subtype key regulators projected onto megena mRNA network modules. (A–F) Node size and label size are proportional to key driver ranking, with text sizes corresponding to higher ranked key regulators. Label color represents direction of subtype‐specific DEGs (BH‐corrected *P* < 0.05) used for key driver prediction, where red corresponds to upregulated DEGs and blue corresponds to downregulated DEGs. (A) S‐immune and the S‐CC‐2 key regulators shown in the immune system process‐enriched module M6. (B) S‐ECM key regulators in the ECM‐enriched module M4. (C) S‐CC key regulators in the DNA packaging‐enriched module M13. (D) S‐LOC key regulators in the RNA binding‐enriched module M8. (E) S‐cilium key regulators in the cilium‐enriched module M15. (F) S‐motif key regulators in the transcription factor targets‐enriched module M11. See also Fig. S7.

To confirm this hypothesis, we performed the key driver analysis (KDA) to identify subtype‐specific key regulators and found that a significant number of subtype‐specific key regulators are also key hub genes in the global megena network (Fig. 4; Fig. S7A–E) [30, 31]. The upregulated DEGs of S‐immune and the downregulated DEGs of S‐CC‐2 are concentrated in the immune response module M6 (Fig. 4A). *CD53*, a tetraspanin protein found primarily on cells of hematopoietic lineage, is the highest ranked key driver in both S‐immune and S‐CC‐2 (Fig. 4A). Other top‐ranked key regulators for S‐immune and S‐CC‐2 such as *SPI1*, *SASH3*, *BIN2*, *PTPRC*, and *FERMT3* are also closely related to myeloid and lymphocyte development, innate immunity, and other hematopoietic cell functions. Together with the estimate scores and DEG enrichment gene signatures (Fig. 2C,E), these key regulators again demonstrate that differences in the amount of external immune cell infiltration are most likely responsible for the differences in immune gene expression for these two subtypes. Though the key regulators of S‐immune are most concentrated in the immune response module (M6) (Fig. 4A), S‐immune is also potentially driven by other key regulators in the major histocompatibility complex (MHC) class I antigen‐processing response module (M7) such as *TAP1*, *TAP2*, and *PSMB9* (Fig. S7F). This finding, combined with the enrichment of the CTL‐high gene signature in S‐immune upregulated DEGs (Fig. 2E), suggests higher levels of antigen‐presentation and cytotoxic T‐cell activation compared with other subtypes.

Key regulators of S‐ECM are uniquely concentrated in the ECM module (M4) (Fig. 4B). Identification of collagen genes *COL5A1*, *COL5A2*, and *COL3A1* as top key regulators points to a tumor microenvironment with significant fibrosis and desmoplastic change. Fibrogenesis in S‐ECM samples is likely due to the presence of an activated cancer‐associated fibroblast (CAF) population in the tumor microenvironment, as indicated by the identification of CAF marker genes as key regulators for S‐ECM, including *FAP*, *ACTA2*, *PDGFRB*, *VCAN*, and *ITGA11* [25, 32, 33, 34] (Fig. 4B). Consequently, the unique upregulation of TGF‐beta family genes *TGFB1*, *TGFBR2*, *TGFBI*, *TGFB1I1*, and *TGFB3* in S‐ECM could be due to increased production of TGF‐beta by CAFs in the tumor microenvironment [33]. Furthermore, CAFs can also secrete other immunosuppressive cytokines such as IL‐6 and C‐C motif chemokine ligand 2, both of which are upregulated in S‐ECM [33].

The key regulators of the rest subtypes are also distinct. S‐CC regulators predicted from both up‐ and downregulated DEGs primarily fall into the DNA packaging and transcription module (M13) and the cell cycle module (M12), such as *PRMT1* and *BCL2L12*, and is suggestive of overactive cell replication in S‐CC (Fig. 4C; Fig. S7G). The key regulators of S‐LOC are primarily concentrated in module M8, which contains a number of genes related to RNA binding and translation (Fig. 4D), and key regulators in S‐cilium predicted by its upregulated DEGs all fall into M15, which is associated with cilium and cilium motility (Fig. 4E). This unique upregulation of cilium‐related genes in S‐cilium could explain the favorable prognosis of this subtype, as the maintenance of normal cilium gene expression has been implicated in decreased tumor proliferation [35]. Lastly, S‐motif key regulators fall into M11, which is enriched for genes containing conserved binding sites for transcription factors such as *SP1*, *ELK1*, and *NRF1* (Fig. 4F). Integrating subtype patient outcome with subtype‐specific key drivers, we can predict appropriate activation or inhibition of key driver gene expression to promote antitumor processes and suppress tumorigenic pathways (Table S3). For example, further upregulation of the S‐immune key regulators will likely improve patient outcome, whereas this is not the case for S‐ECM and S‐CC‐2 (Table S3).

We found that the impact of differentially expressed (DE‐) miRNAs on gene expression varies by subtype. S‐ECM and S‐CC‐2 have the largest number of DE‐miRNAs compared with the other subtypes, whereas S‐CC, S‐LOC, and S‐cilium each have fewer than 12 DE‐miRNAs and consequently less miRNAs influence (Tables S4 and S5). DE‐miRNAs also seem to be significantly correlated with subtype‐specific key regulator expression (Figs S1A,B and S2). Subtype‐specific methylation analysis showed that cis‐differential methylated probe (DMP) genes also include a large portion of subtype‐specific key regulators, especially those that are predicted to downregulate DEGs in the S‐cilium and S‐motif (Fig. S1C,D, Table S6). Trans‐DMP genes are associated with upregulation of cell cycle signal in S‐CC‐2, as well as downregulation of ECM genes in S‐immune (Fig. S1E,F). Finally, we identified subtype‐specific CNAs using gistic2.0 [36], which produced different patterns of genomic locations with copy number variations for each subtype and predicted many more deletions than amplifications for all the subtypes (Figs S1G–J, S8 and S9, Tables S4 and S7). Most of the common gene amplifications and deletions involved in HGSOC tumorigenesis are simultaneously present in most subtypes and not subtype‐specific, except *AKT1/2* amplifications and *BRCA1/2* deletions, which are present in only two subtypes (Table S8).

### Subtype‐specific therapeutics

**Fig. 5:**
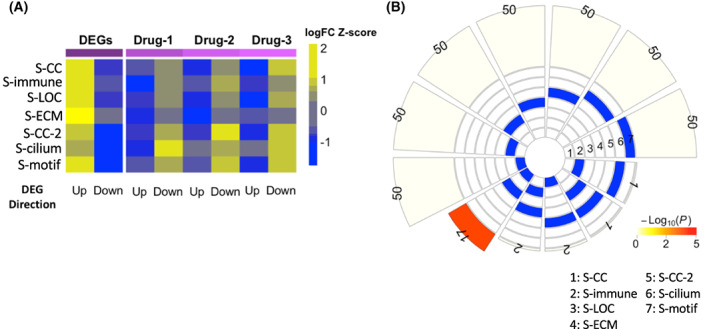
Characterization of the top predicted drugs for each of the seven HGSOC subtype. (A) Heatmap of the average log_2_(fold change) values of the top 50 DEGs (ranked by *P*‐value) for each subtype versus the normal ovarian tissues and those of the same set of genes induced by the top 3 predicted drugs for the respective subtype. The log_2_(fold change) values are *z*‐score normalized within each subtype. (B) Intersection of the lists of top predicted drugs for the seven subtypes. Here, we considered the top 50 drugs for each subtype. Intersections with at least one shared drug are shown. Intersection significance was assessed by FET. Color gradient shows −log_10_(FET *P*‐value).

The genetic heterogeneity of the seven HGSOC subtypes suggests the need for individualized treatment strategies. To this end, we used the recently developed Ensemble of Multiple Drug Repositioning Approaches (emudra) to identify drugs that can reverse differentially expressed gene signatures in each subtype in comparison with normal ovarian tissues [37] (see Methods). DEGs between subtypes versus normal samples showed significant and coherent functional categories that are largely similar to those from DEGs identified by comparing subtypes to the rest of the tumor samples (Table S9). Additional input to emudra included drug perturbation data from over 10 000 compounds tested on four cancer cell lines [37]. Comparison of the log_2_(fold change) values of the DEGs between each subtype versus the normal samples to those of the drug treatment DEGs shows that the top‐ranked drugs for each subtype can reverse the DEG signature of the subtype (Fig. 5A; Table S9).

The unique gene expression profiles of the HGSOC subtypes resulted in small overlap between the predicted drug lists (Fig. 5B). Among all the 21 pairwise comparison, 2 pairs of subtypes share 1 drug, 2 pairs share 2 drugs, and a pair (S‐CC and S‐LOC) share 17 drugs. The large overlap between S‐CC and S‐LOC may be due to relatively similar expression profiles though a vast majority of their drugs are different. Although the DEGs upregulated in S‐CC, S‐LOC, and S‐CC‐2 are all enriched for cell cycle‐related processes (Table S9), the predicted top drugs are very different (Tables S2 and S10). Subtypes with similarly good or poor prognoses also have different drugs. Although topoisomerase inhibitors have been shown to be effective in treating platinum‐resistant HGSOC, they are predicted to reverse only S‐CC‐2 DEG signatures but not those for S‐ECM (Table 3) [38]. Similarly, the top‐ranked drugs for S‐immune and S‐cilium, two subtypes with good prognoses, belong to different drug classes as well (Table 3, Table S10).

**Table 3:** Summary of top‐ranked repositioned drugs with available annotation information.

Subtype	Rank	Compound	Mode of action	Target(s)	Disease area	Phase
S‐CC	2	Tyrphostin ag 1478	EGFR inhibitor	EGFR, MAPK14		Preclinical
	4	bms‐754807	IGF‐1 inhibitor	AKT1, IGF1R		Phase 2
	11	4‐Demethoxy‐daunorubicin	Topoisomerase inhibitor	TOP2A	Hematologic malignancy	Launched
S‐immune	10	Barasertib	Aurora kinase inhibitor	AURKA, AURKB		Phase 2 Phase 3
	15	BRD‐K27305650	mTOR inhibitor, PI3K inhibitor, DNA‐dependent protein kinase inhibitor, phosphodiesterase inhibitor, PLK inhibitor	AKT1, CHEK1, GSK3B, LCK, MAPK1, MAPK11‐12, MAPK14, MAPK8, MTOR, PIK3CA‐B, PIK3CD, PIK3CG, PLK1, PRKCA, PRKDC, ROCK1, RPS6KB1, SGK1		Preclinical
S‐LOC	5	azd8055	mTOR inhibitor	MTOR		Phase 1
	7	BRD‐K44432556	Hypoxia‐inducible factor activator	HIF1A		Preclinical
	8	BRD‐K57080016	MEK inhibitor	MAP2K1		Phase 3
S‐ECM	7	Ropinirole	Dopamine receptor agonist	ADRA2A‐C, DRD1‐5, HTR1A‐B, HTR2A‐D	Neurology, psychiatry	Launched
	12	Alclometasone	Glucocorticoid receptor agonist	CYP3A4, NR3C1, SERPINA6	Dermatology	Launched
	30	Toremifene‐citrate	Estrogen receptor antagonist, selective estrogen receptor modulator (SERM)	ESR1	Oncology	Launched
S‐CC‐2	3	Camptothecin	Topoisomerase inhibitor	TOP1		Phase 3
	13	Doxorubicin	Topoisomerase inhibitor	TOP2A	Hematologic malignancy, oncology	Launched
	16	4‐Demethoxy‐daunorubicin	Topoisomerase inhibitor	TOP2A	Hematologic malignancy	Launched
S‐cilium	14	mdl 11,939	Serotonin receptor antagonist	HTR2A‐C		Phase 2
	32	tcs 359	FLT3 inhibitor	FLT3		Preclinical
S‐motif	6	Prilocaine	Local anesthetic	SCN1‐5A, SCN7‐11A	Neurology, psychiatry	Launched
	17	BRD‐A59174698	Adrenergic receptor agonist	ADRB2	Obstetrics, gynecology	Launched
	33	Atovaquone	Mitochondrial electron transport inhibitor	DHODH	Infectious disease	Launched

## Discussion

In this study, we developed a novel clustering algorithm called MWINA and applied it to the multiomic data from HGSOC to uncover seven novel HGSOC subtypes that are highly distinct in OS. These subtypes are robust and not biased toward clinical parameters such as tumor stage and grade. More importantly, the subtypes are comprehensively characterized by specific molecular changes involving microRNAs, DNA methylation and copy number alterations as well as molecular networks.

By utilizing MWINA, our study identified subtypes with coherent gene expression signatures that can be categorized into very poor (S‐ECM and S‐CC‐2), intermediate (S‐CC and S‐motif), and relatively good (S‐immune, S‐LOC, and S‐cilium) prognoses [14, 39]. In addition, our multiscale co‐expression network analysis of HGSOC and the extension into multiomic correlation networks enabled in‐depth, comprehensive characterization of the identified subtypes. Previous work on ovarian cancer subtyping such as Zhang et al.'s subtype analysis showed a concentration of both high‐grade and late‐stage tumors in their subtype‐2, which also demonstrated enrichment in ECM‐ and EMT‐related genes [14]. However, even though the subtype S‐ECM from this study is also enriched for similar genes, there is no significant difference in tumor grade and stage among the MWINA subtypes. Furthermore, the other MWINA subtypes also have clearly defined molecular signatures, such as immune system activation (S‐immune), cell cycle (S‐CC and S‐CC‐2), cilium (S‐cilium), and transcription factor binding motifs (S‐motif) that are associated with prognostic outcome. Although the subtypes from Zhang et al. had significantly different overall survival times, it is unclear whether the other subtypes besides subtype‐2 had well‐defined gene signatures and key regulators that are associated with prognosis. Furthermore, the highly translational nature of our work in the utilization of emudra to predict subtype‐specific therapies lays the foundation for the development of subtype‐based personalized treatment strategies in HGSOC.

Interestingly, two subtypes with similar ages at diagnosis and similarly favorable prognoses are regulated by completely different molecular pathways. The tumors from S‐immune display high immune cell infiltrates and low stromal cell infiltrates as shown by their DEG signatures and estimate profiles. By contrast, the tumors from S‐cilium have very low immune gene expression, and while their ECM gene expression is also low, they display an upregulated cilium gene signature that could point to the S‐cilium tumors being in a quiescent state, resulting in longer OS [40, 41, 42, 43]. However, loss of cilia expression, such as observed in patients belonging to S‐ECM, leads to aberrant hedgehog (Hh) signaling in HGSOC, resulting in maintenance of cancer stem cells, decreased cell differentiation, and eventual cancer progression [35]. This may open the opportunity for subtype‐specific treatment with ciliogenesis‐promoting drugs such as glucocorticoids, fibrates, and other nuclear receptor modulators [44]. Alclometasone, a glucocorticoid receptor agonist and one of the top‐ranked repositioned drugs for S‐ECM, could prove to be highly effective for patients belonging to this subtype. On the contrary, the patients from S‐immune may benefit from immune checkpoint inhibitors combined with mTOR inhibitors to enhance the activation and cytotoxicity of the relatively large amount of CD8+ T‐cells already present in this subtype [45, 46, 47]. BRD‐K27305650, an mTOR inhibitor predicted to reverse S‐immune gene expression signatures, could be an ideal candidate for this purpose.

Besides the loss of cilia expression, S‐ECM also has upregulated ECM and immune response which are under the regulation of microRNAs and methylations. On the contrary, S‐CC‐2, which has similarly poor survival as S‐ECM, is characterized by a gene signature profile that is almost completely opposite. The patients in S‐CC‐2 have downregulated ECM and immune response as well as upregulated cell cycle‐related process also under significant regulation of microRNAs and methylations. It is interesting to note that although S‐CC is similar to S‐CC‐2 in both tumor cell type composition and upregulated cell cycle gene expression, the patients in S‐CC have longer OS. This difference in survival may be due to miRNA and methylation dysregulation seen in S‐CC‐2, as S‐CC has only one‐sixth of DE‐miRNAs and fewer than 2.5% of DMPs compared with the Subtype‐5.

It has been shown that expression of various ECM‐remodeling proteins and components is associated with poor prognosis in HGSOC [22]. Similar to the mesenchymal subtype from the original TCGA study of ovarian cancer, S‐ECM shows upregulation of genes such as *COL5A2*, *COL1A1*, *COL3A1*, *THBS2*, *COL11A1*, *COL6A3*, and *MMP2* that are involved in epithelial‐to‐mesenchymal transformation (EMT) processes, metastasis, and chemoresistance [48, 49, 50, 51, 52]. The lack of significant enrichment in interferon signaling combined with high stromal cell infiltration in S‐ECM could also contribute to chemoresistance, as interferon‐gamma derived from CD8+ lymphocytes is involved in modulation of fibroblast‐associated resistance to platinum‐based chemotherapy [53].

Ropinirole, a dopamine receptor agonist predicted to reverse the gene signatures of S‐ECM, may be an effective innovative therapy for targeting the TGF‐beta and EMT processes. A previous study showed that defects in the dopamine D2 receptor (D2R) led to a pro‐inflammatory environment characterized by increased tumor necrosis factor‐alpha, TGF‐beta1, Smad3, Snail1, as well as increased EMT processes and increased production of ECM proteins such as vimentin, fibronectin 1, and collagen I, and an increase in normal D2R subsequently downregulated the expression of these proteins [54]. Similarly, dopamine was also found to improve the survival of mice implanted with liver cancer [55].

Differences in gene expression signatures and survival outcomes between the younger S‐immune and the older S‐CC‐2 patients are suggestive of the tumor evolution trajectory of HGSOC [56]. Earlier stage of HGSOC development is often characterized by greater lymphocyte infiltration that leads to an active immune response, whereas cancer later in the evolutionary trajectory demonstrates negligible immune cell presence in the tumor microenvironment [56]. This phenomenon was characterized previously by the TCGA group in their identification of the immunoreactive subtype, which has many resemblances to S‐immune [5]. Therefore, it is not surprising that the key regulators downregulated in S‐CC‐2, but upregulated in the S‐immune include *CD53*, a key player in B‐cell activation [57, 58], and *PTPRC*, a regulator in T‐cell and B‐cell antigen receptor signaling and positive regulator of T‐cell activation [59]. Additionally, various types of interferon signaling, which is downregulated in S‐CC‐2 but upregulated in S‐immune, have been shown to deter HGSOC tumor progression and improve patient prognosis [60, 61, 62]. Specifically, increased type I interferon signaling reduces immunosuppression in the tumor microenvironment by upregulating CD45+ immune cells, CD8+ T‐cells, and natural killer cells, and is associated with favorable outcomes [45, 63, 64]. This explains the longer survival seen in the patients from S‐immune, which has significantly higher expression of tumor infiltrating lymphocytes (TILs) marker compared with other subtypes [65, 66]. *PRMT1* and *BCL2L12*, the top two upregulated regulators of S‐CC, also play critical roles in tumor development. *BCL2L12* can bind to p53 in the nucleus to prevent association with p53 targets [67] and also acts as anti‐apoptotic factor in glioblastoma by inhibiting caspases 3 and 7 in the cytoplasm [68, 69, 70]. *PRMT1* expression is critical in maintaining cell cycle processes in many types of cancer, and its depletion significantly reduces the number of cells in the S phase of the cell cycle [71].

Our innovative multiomics‐based approach to patient clustering in HGSOC yielded seven molecular subtypes that have distinct signatures in gene expression and upstream regulation not observed in previous studies. In addition to the subtypes with significantly distinct OS, it is interesting that we identified two pairs of subtypes with similar prognostic outcomes but entirely opposite gene signatures as well as different drug susceptibility profiles. These findings highlight the heterogeneity of the disease even among patients that have almost identical clinical parameters and demonstrate the critical importance of accurate and biologically meaningful subtypes in HGSOC.

However, there are a few important limitations to our study. Although we categorized HGSOC patients into subtypes with distinct molecular signatures, the bulk nature of the RNA‐seq data means it is difficult to attribute upregulated genes to the tumor cells alone. Since it is highly likely that gene signatures are also reflective of stromal and immune cell infiltration, it may be necessary to look into single‐cell sequencing data in the future in order to refine our drug repositioning results to target both tumor and stromal subsets simultaneously. In addition, it is important to recognize that the study of ovarian cancer is gradually shifting away from an era of placing tumors into discrete subtypes and to modeling HGSOC subtypes as various stages in the tumor development trajectory [72]. Geistlinger et al. concluded that current HGSOC subtypes may be reflective of the later, subclonal stages of the disease, and subtype‐specific therapies should instead target genomic alterations that occur earlier in tumor evolution in order to be effective.

In summary, we integrated large‐scale multiomics data to identify seven HGSOC subtypes which not only have significantly distinct overall survival but also carry unique patterns of gene expression, microRNA expression, DNA methylation, and copy number alterations. These subtypes show more significant prognostic difference. Second, we performed multiscale gene co‐expression network analysis to identify subtype‐specific key regulators and predicted targeted therapies based on subtype‐specific gene expression patterns. While the previous studies have done subtyping analysis of HGSOC, our study is the first one to integrate three major components including subtyping analysis, network analysis, and drug repositioning. Additional work is needed to translate this basic research into clinical applications but the more precise molecular subtyping, the comprehensive depiction of the molecular features of these subtypes, and the predicted subtype‐specific therapies derived from this study lay down a solid foundation for precision medicine for HGSOC. Future work will include experimental validation of subtype‐specific pathways and regulators, predicted therapeutics, as well as creating a biomarker panel of top subtype‐specific key regulators that can distinguish patient subtypes in a clinical setting.

## Methods

### Data processing

Level 3 open access data available through the NIH National Cancer Institute Genomic Data Commons (GDC) Data Portal for the Cancer Genome Atlas (TCGA) ovarian serous cystadenocarcinoma mRNA expression (Illumina HiSeq RNA‐Seq and HG‐U133A microarray) and miRNA expression (Illumina HiSeq miRNA‐Seq, RPKM data) were downloaded and processed in November 2014, and methylation beta values (Illumina Infinium HumanMethylation27 array) and masked copy number segment data (Affymetrix SNP 6.0 array) were downloaded and processed in August 2018. Clinical data, including stage, residual tumor size, tumor grade, and OS, were also obtained through the GDC Data Portal. OS is a closed dataset and is defined as time to death from day of diagnosis for deceased patients or time to last follow‐up for living patients at the time of data collection.

Genes with no or low expression in RNA‐Seq mRNA (*n* = 420) and miRNA (*n* = 486) expression data were removed. The filtered data then underwent log_2_ transformation, quantile normalization, and correction by linear regression for confounding covariate effects including batch, tissue source site, center, plate, race, and age. Genes with no or low expression in the microarray gene expression data for normal (*n* = 8) and tumor (*n* = 586) samples were also filtered out, and log_2_ transformation, quantile normalization, and correction by linear regression for confounding covariate effects including batch, tissue source site, center, plate, race, and age were subsequently performed. 18 243 mRNA genes and 705 miRNAs from RNA‐Seq data remained after filtering, and 12 042 genes from microarray data remained after filtering. These remaining genes were used for downstream analyses. Five hundred and twelve patients with mRNA expression, microRNA expression, methylation, and CNA data were used for subtype identification. Though the RNA‐Seq data were available for only 420 samples and 486 samples for mRNA and miRNA expression, there is no significant difference in the clinical demographics between the 512 patients used for subtype clustering and the sample subset with RNA‐Seq data (Table S2). Additionally, all the subtypes were well‐represented, with 70–90% of patients from each subtype having mRNA RNA‐Seq data available, and 86–97% of patients from each subtype having miRNA RNA‐Seq data available.

Methylation beta values were converted to *M*‐values using the function beta2m from the lumi R package [73], and then the *M*‐values were corrected by linear regression for confounding covariate effects including batch, tissue source site, center, plate, race, and age. Only corrected *M*‐values from primary tumor samples (*n* = 540) were used for differential methylated probe (DMP) analysis and DMP‐gene correlation analysis. Copy number segment data did not undergo normalization or covariate correction, since the data had been previously tangent normalized as part of the data preparation and analysis pipeline from the National Cancer Institute Genomic Data Commons (https://docs.gdc.cancer.gov/Data/Bioinformatics_Pipelines/CNV_Pipeline/). Briefly, the segment data were normalized by subtracting variations found in a set of normal samples, and only segment data from primary tumor samples (*n* = 568) were used for downstream analyses.

### MWINA clustering for *de novo* subtype identification

Multiscale Weighted Interaction Network Analysis (MWINA) was used to create *de novo* subtype clusters using a set of survival‐associated multiomic features described previously [20]. A combination of features including mRNA expression, microRNA expression, copy number alteration, and DNA methylation from TCGA high‐grade ovarian serous adenocarcinoma was selected based on univariate Cox proportional hazard model analysis (*P* < 0.05) [14]. A total of 4526 features were used, including the expression of 1651 mRNA genes, 140 microRNAs, 2191 somatic copy number alterations, and 455 DNA methylation sites [14]. Features were normalized as follows:

$${\text{Feature}}_{\mathrm{norm}}=\frac{\text{Feature}-{\text{Median}}_{\text{controls}}}{{\mathrm{SD}}_{\text{patients}}},$$

where Feature_norm_ is the normalized expression value, Feature is the unnormalized expression value, Median_controls_ is the feature median of expression of the normal control samples, and SD_patients_ is the feature standard deviation of the patient samples [14].

MWINA employs multiresolution optimization of Newman's modularity (denoted *Q*) with Reichardt‐Bornholdt (RB) parameter [21], γ_RB_, allowing for detection of fine‐resolution modules with small γ_RB_ <1, coarse‐grained modules with larger γ_RB_ >1, and converging to Q when γ_RB_ = 1. In order to optimize *Q*(γ_RB_ = γ′) for some γ′ > 0, we employed an iterative three‐step optimization approach:

1. Split: For each module, perform *k*‐median clustering on the shortest path distance (SPD) for *k* = 2, …, *k*_max_ for some *k*_max_, where the solution with *k* = *k*′ optimizing *Q*(γ_RB_ = γ′) is chosen.
2. Merge: For all pairs of modules after step 1, perform the following iterative procedure.
  - Identify an adjacent module pair which improves *Q*(γ_RB_ = γ′) the most and merge if the merged *Q*(γ_RB_ = γ′) is higher than the preceding solution,
  - Repeat step 2‐a until no further pair is found.
3. Refine: Perform extremal optimization by assigning node fitness as a function of *Q*(γ_RB_ = γ′), shifting module membership of least fit nodes.

Steps 1–3 are repeated iteratively till no further improvements are made.

With the aforementioned algorithm, solutions are obtained for γ_min_ ≤ γ′ ≤ γ_max_ with some increment dγ. Let us denote the solution for each γ′ as *Z*(γ′). In order to identify similar solutions and range of γ′ that behave similarly, adjusted Rand Index (ARI) was employed to measure similarity between two distinct solutions *Z*(γ′) and *Z*(γ″) with some threshold of ARI > ARI_cutoff_ to determine similar. For each = {γ|ARI(*Z*(γ_a_), *Z*(γ_b_)) > ARI_cutoff_ for γ_a_, γ_b_ ɛ}, the median solution showing the largest overall score. The median solutions across all are gathered to identify the final multiscale solution of MWINA.

### Chi‐squared test for comparing overall survival

The chi‐squared statistic was computed to compare the subtype OS for the MWINA TCGA‐HGSOC subtypes, with the null hypothesis being that there is no significant difference among the distributions of the subtype OS.

### Cluster consensus and robustness evaluation

Subtype consensus was evaluated by performing MWINA clustering 50 times, each time using 80% of randomly sampled features and 80% of randomly sampled patients. Results from the 50 times bootstrapping were summarized into a *n*‐by‐*n* matrix of pairwise sample concordance values, where *n* is the total number of patients, and the value at position *n*_*ij*_ is the probability that patient *i* and patient *j* are in the same cluster:

$$n_{\mathrm{ij}}=\frac{\#\text{clusters containing patients}\;i\;\text{and}\;j}{\#\text{bootstrapping}}.$$

Individual subtype robustness was evaluated by calculating the probability that the sum of pairwise concordance values for patients within a subtype is greater than the sum of pairwise concordances for the same number of randomly sampled patients. Using this probability, we calculated a robustness empirical *P*‐value for each subtype based on the comparison of the sum of subtype patient pairwise concordances to that of 1 million iterations of randomly sampled patients:

$$\text{empirical}\;P\;\text{value}=1-P\left(\frac{\sum P\left(\text{consensus}|\text{cluster}\right)}{\text{length}\left(\text{cluster}\right)}>\frac{\sum P\left(\text{consensus|random}\right)}{\text{length}\left(\text{cluster}\right)}\right).$$

### Differential gene expression analysis

Differentially expressed genes (DEGs), miRNAs (DE‐miRNAs), and methylated probes (DMPs) were identified using expression data from TCGA (as described previously) with the limma R package [74]. Subtype‐specific DEGs were found between samples within each subtype versus remaining samples from all other subtypes and also between subtype samples versus samples with normal ovarian tissue. HG‐U133A microarray data were used for DEG analysis between tumor samples versus normal tissue, since there are no RNA‐Seq data available through TCGA for normal tissue. DE‐miRNAs and DMPs were calculated between samples within each subtype versus remaining samples from all other subtypes. Significantly differentially expressed genes were defined as having fold change (FC) ≥ 1.2 and Benjamini–Hochberg (BH)‐adjusted *P* < 0.05.

### Fisher's exact test

Fisher's exact test (FET) was performed to evaluate gene set overlaps between MSigDB annotated gene sets, subtype gene sets (such as subtype‐specific DEGs, DE‐miRNA‐affected genes, cis‐eDMP and trans‐eDMP genes, and key regulators), megena module genes, and other gene sets including cancer‐associated fibroblast, cytotoxic T‐cell, and myeloid‐derived suppressor cell genes signatures. FET was also performed to determine overlap between TCGA‐HGSOC subtype samples for MWINA subtypes and Zhang et al. [14] subtypes. FET significance is defined by *P*‐value < 0.05 after Bonferroni correction.

### Gene set enrichment

Gene set annotations used in gene set enrichment analysis were downloaded from the Molecular Signatures Database (MSigDB) [75, 76, 77]. One‐sided Fisher's exact test (FET) was performed between MSigDB gene sets versus subtype DEGs, miRNA‐correlated genes, genes under methylation regulation, and predicted amplified or deleted genes.

### megena network construction

megena (Multiscale Embedded Gene Co‐expression Network Analysis) is a novel gene co‐expression network framework that utilizes parallelized embedded network construction and novel clustering techniques to identify multiscale modules in Planar Filtered Networks (PFNs) [29]. The megena gene co‐expression network for HGSOC was constructed RNA‐Seq mRNA expression data from the GDC Data Portal and processed as described previously. A false discovery rate (FDR) threshold of 0.10 was used to filter out insignificant gene–gene interactions, and a module FDR threshold of 0.05 was applied to identify significant multiscale gene modules.

### Key driver analysis

Key driver analysis (KDA) was performed for to identify subtype‐specific key regulators (drivers). The global HGSOC megena gene co‐expression network (N) and significant subtype‐specific DEGs (G) (between subtype samples versus remaining tumor‐bearing patient samples from all other subtypes, as described above) were used as inputs for KDA. Fisher's exact test was used to identify network nodes whose one‐ to two‐degree neighbors are enriched in genes in G, and nodes with a BH‐corrected *P*‐value < 0.05 are identified as key regulators (drivers). This analysis is done separately for up and downregulated DEGs for all seven HGSOC subtypes to identify upregulated and downregulated key regulators.

### Correlation analysis

Spearman correlation was performed to identify mRNA‐mRNA, mRNA‐miRNA, and mRNA‐methylation relationships. Pearson correlation was performed between patient age at diagnosis and OS time and between estimate tumor purity scores and OS time. For features with different numbers of samples, the intersection of samples was used. Correlation significance is defined by *P*‐value < 0.05 after Bonferroni correction.

### Prediction of subtype amplified and deleted genes

TCGA patient copy number segment data were categorized by subtype membership and genes with significant copy number altered regions for each subtype were identified using the gistic2.0 algorithm available via the genepattern notebook online tool suite with default settings [36, 78]. Human genome assembly GRCh38 (hg38) was used as the *refgene* file. The marker file was created from the TCGA segmentation file and records the start and end positions of each segment as individual markers.

### Drug repositioning

Subtype‐specific DEGs between subtype samples versus normal ovarian tissue samples were identified as described previously. We used more relaxed cutoffs to determine DEGs for each subtype due to a small number (8) of normal samples curated in the TCGA cohort. For subtypes 4 and 5, we used *P*‐value < 0.15, and for the rest subtypes, we used *P*‐value < 0.2. These DEG signatures were taken as input for the Ensemble of Multiple Drug Repositioning Approach (emudra) as described previously [37] to identify drugs that can reverse these signatures. We considered only the data from 4 cell lines (MCF7, HT29, A375, and A549) treated with the largest number of drugs in the LINCS cohort [79]. Specifically, 12 812, 13 622, 12 312, and 13 159 drugs were tested in the MCF7, HT29, A375, and A549 cell lines, respectively, while 8764 drugs tested in all four cell lines. Only the drugs tested in at least two of the four cell lines were included in the final ranking.

We obtained the annotations of the compounds from the Repurposing Hub drug repurposing hub (https://clue.io/repurposing‐app). The Repurposing Hub contains comprehensive annotations for a total of 6125 compounds: 2369 Launched drugs, 1619 drugs that reached phases 1–3 of clinical development, 96 compounds that were previously approved but withdrawn from use, and 2041 preclinical or tool compounds. Specifically, we extracted a variety of drug information, including compound name, clinical trial status, mechanism of action, protein targets, disease areas, and approved indications (where applicable). We excluded the compounds that have no annotation.

### Quantification and statistical analysis

Statistical details and parameters including the exact value of *n*, the definition of center, dispersion and precision measures (mean ± SD) and statistical significance are reported in the figures and figure legends. The data are judged to be statistically significant if *P* < 0.05 after Bonferroni correction for correlation analysis and FET, or fold change ≥ 1.2 and *P* < 0.05 after Benjamini–Hochberg correction for differential expression analysis. Survival significance for MWINA subtypes is determined by the chi‐squared test. Differences in sample features and sample clinical data were determined by the Kruskal–Wallis analysis of variance test and Student's *t*‐test, and significance was reached if *P* < 0.05. Statistical analysis was performed with R.

## Conflict of interest

The authors declare no conflict of interest.

## Author contributions

BZ conceptualized and designed the study. MJW, S‐HC and YAW participated in the study design. MJW and S‐HC participated in the discussion of the results. YAW performed research; RN, WS, XZ and SV contributed software tools and participated in data analysis and interpretation. YAW and BZ wrote the manuscript. All the authors reviewed and revised the paper.

## Supporting information


**Fig. S1.** Functional annotation of upstream regulator signatures of the HGSOC subtypes. (A‐B) Heatmaps showing enrichment of MSigDB gene sets in (A) upregulated genes and (B) downregulated genes for each set of subtype DE‐miRNA‐affected genes. DE‐miRNAs were identified by comparing the samples in a subtype with the rest of the samples. (C‐D) Heatmaps showing enrichment of MSigDB gene sets in the subtype cis‐eDMP genes for (C) hypermethylated and (D) hypomethylated DMPs. (E‐F) Heatmaps showing enrichment of MSigDB gene sets in genes (E) upregulated and (F) downregulated for each subtype that are also predicted to be under trans‐regulation by subtype DMPs. DMPs were identified by comparing the samples in each subtype with the rest of the samples. (G‐H) Heatmaps showing enrichment of MSigDB gene sets in the predicted (G) amplified and (H) deleted genes for each subtype. (I‐J) Bar plots showing number of overlapping (I) amplified and (J) deleted genes between subtype pairs. Green dots below each bar indicate the subtype pairs being compared.
**Fig. S2.** Subtype‐specific DE‐miRNAs and DMPs and their relationships with megena network modules. Graphical representation of regulation of subtype‐specific DEGs by top 10 subtype DE‐miRNAs and DMPs. DEGs are highlighted by color within megena network modules, and DEGs labeled in white are also subtype key regulators. DE‐miRNAs and DMPs are also color coded as shown. Edges between megena module nodes and DE‐miRNAs and DMPs show significant positive (red) or negative (blue) expression correlations. (A) S‐immune DE‐miRNAs and DMPs and the immune system process‐enriched module M6. (B) S‐ECM DE‐miRNAs and DMPs and the ECM‐enriched module M4. (C) S‐CC‐2 DE‐miRNAs and DMPs and the immune system process‐enriched module M6.
**Fig. S3.** MWINA subtypes are robust redistributions of Zhang et al HGSOC subtypes and have some differences in age at diagnoses. (A) Plots of HGSOC sample concordances. Samples are along the x and y axes, and color intensities correspond to sample pairwise concordance probabilities. Concordance probabilities range from 1.0 along the diagonal (red) to 0 (white). Concordance clustering is based on Euclidean, Manhattan, or Maximum distance calculation methods. (B) Heatmap of ‐log10(p‐value) from Student's t‐test for age at diagnosis between subtype pairs. Only comparisons between Group 4 or Group 5 versus other subtypes were significant (p < 0.05). (C) Heatmap of ‐log10(p‐value) from Fisher's exact test for overlap between patients from MWINA subtypes and patients from Zhang et al (2013) subtypes.
**Fig. S4.** HGSOC subtypes with similar prognosis have distinct gene expression signatures. (A‐B) Heatmap of MSigDB gene set enrichment for overexpressed (A) and underexpressed (B) genes in HGSOC tumor samples of each subtype with respect to normal controls. Enrichment is represented by FET ‐log10(BH‐adjusted p‐value) for gene set overlap. (C‐F) Overlap of upregulated or downregulated DEGs between (C) poor surviving subtypes (S‐ECM and S‐CC‐2) and (D‐F) favorable survival subtypes (S‐immune, S‐LOC, and S‐cilium). P‐value is calculated from Fisher's exact test (FET), and nonsignificant FET p‐values (p > 0.05) are not shown. (G‐H) Heatmaps showing subtype DEG enrichment for genes up‐ or downregulated in (D) CAFs from ovarian tumors compared with normal ovarian stroma, and for genes upregulated in (E) MDSCs (compared with PMNs) from NSCLC. Enrichment is determined via FET. Color gradient shows ‐log10(FET p‐value). FET, Fisher's exact test; CAF, cancer‐associated fibroblast; MDSC, myeloid‐derived suppressor cell; NSCLC, non‐small‐cell lung cancer. Related to Fig. 2.
**Fig. S5.** megena module genes are enriched for HGSOC subtype upregulated DEGs. Sunburst plots showing overlap of subtype‐specific upregulated DEGs with megena network module genes. Colors correspond to ‐log10(FET p‐value). Modules with p‐value >0.05 are not colored. Related to Fig. 3.
**Fig. S6.** megena module genes are enriched for HGSOC subtype downregulated DEGs. Sunburst plots showing overlap of subtype‐specific downregulated DEGs with megena network module genes. Colors correspond to ‐log10(FET p‐value). Modules with p‐value >0.05 are not colored. Related to Fig. 3.
**Fig. S7.** Overlap of subtype‐specific key driver genes and megena module genes. (A‐E) Overlap of subtype‐specific key driver genes and megena module hub genes for (A) S‐CC and cell cycle‐enriched module M83, (B) S‐immune and immune system process‐enriched module M6 and interferon response‐enriched module M7, (C) S‐ECM and ECM‐enriched module M4, (D) S‐CC‐2 and immune system process‐enriched module M6 and cell cycle‐enriched module M12, and (E) S‐cilium and cilium‐enriched module M15. P‐value is calculated from Fisher's exact test (FET) ECM, extracellular matrix; FET, Fisher's exact test. (F‐G) Subtype‐specific key drivers shown in megena modules. Node size and label size are proportional to key driver ranking, with larger node and text sizes corresponding to higher ranked key drivers. Label color represents direction of subtype‐specific DEGs (p < 0.05) used for key driver prediction, where red corresponds to upregulated DEGs and blue corresponds to downregulated DEGs. (F) S‐immune key drivers shown in the interferon response‐enriched module M7. (G) S‐CC key drivers in the cell cycle‐enriched module M12. Related to Fig. 4.
**Fig. S8.** Focal somatic copy number alterations for subtype samples by chromosome location of amplified regions
**Fig. S9.** Focal somatic copy number alterations for subtype samples by chromosomal location for deleted regions.
**Table S1.** Kruskal–Wallis test for significance of clinical traits and estimate scores in subtypes.
**Table S2.** Student's t‐test of clinical traits between samples with RNA‐Seq data and samples used for subtype identification.
**Table S3.** Predicted therapeutic actions on subtype‐specific key driver genes.
**Table S4.** Number of differentially expressed genes, miRNAs, methylated positions, and amplifications and deletions for each subtype compared with the rest of the samples.
**Table S5.** Overlap between subtype DEGs or key drivers and genes significantly correlated (Spearman p < 0.05) with subtype DE‐miRNAs.
**Table S6.** Overlap between subtype DEGs or key drivers and subtype cis‐eDMP genes.
**Table S7.** Overlap between subtype DEGs or key drivers and predicted amplified or deleted genes.
**Table S8.** Presence of amplified and deleted genes commonly associated with HGSOC tumorigenesis for all subtypes.
**Table S9.** Functional annotation enrichment of DEGs between subtype vs. normal ovarian tissue used for drug repositioning analysis.
**Table S10.** Top 5 repositioned drugs for each subtype.

## Data Availability

Input data for MWINA clustering have been deposited in Mendeley Data at https://doi.org/10.17632/67yzwc826b.2. megena R package is available at CRan (https://cran.r‐project.org/).
