# Supplementary material for: Multi‐omics‐based analysis of high grade serous ovarian cancer subtypes reveals distinct molecular processes linked to patient prognosis
Source: FEBS Open Bio. 2023 Feb 24;13(4):617–37. doi: 10.1002/2211-5463.13553 (PMC10068328; doi:10.1002/2211-5463.13553)
Supplement: Supplementary file 1 — Fig. S1. Functional annotation of upstream regulator signatures of the HGSOC subtypes. (A‐B) Heatmaps showing enrichment of MSigDB gene sets in (A) upregulated genes and (B) downregulated genes for each set of subtype DE‐miRNA‐affected genes. DE‐miRNAs were identified by comparing the samples in a subtype with the rest of the samples. (C‐D) Heatmaps showing enrichment of MSigDB gene sets in the subtype cis‐eDMP genes for (C) hypermethylated and (D) hypomethylated DMPs. (E‐F) Heatmaps showing enrichment of MSigDB gene sets in genes (E) upregulated and (F) downregulated for each subtype that are also predicted to be under trans‐regulation by subtype DMPs. DMPs were identified by comparing the samples in each subtype with the rest of the samples. (G‐H) Heatmaps showing enrichment of MSigDB gene sets in the predicted (G) amplified and (H) deleted genes for each subtype. (I‐J) Bar plots showing number of overlapping (I) amplified and (J) deleted genes between subtype pairs. Green dots below each bar indicate the subtype pairs being compared. Fig. S2. Subtype‐specific DE‐miRNAs and DMPs and their relationships with megena network modules. Graphical representation of regulation of subtype‐specific DEGs by top 10 subtype DE‐miRNAs and DMPs. DEGs are highlighted by color within megena network modules, and DEGs labeled in white are also subtype key regulators. DE‐miRNAs and DMPs are also color coded as shown. Edges between megena module nodes and DE‐miRNAs and DMPs show significant positive (red) or negative (blue) expression correlations. (A) S‐immune DE‐miRNAs and DMPs and the immune system process‐enriched module M6. (B) S‐ECM DE‐miRNAs and DMPs and the ECM‐enriched module M4. (C) S‐CC‐2 DE‐miRNAs and DMPs and the immune system process‐enriched module M6. Fig. S3. MWINA subtypes are robust redistributions of Zhang et al HGSOC subtypes and have some differences in age at diagnoses. (A) Plots of HGSOC sample concordances. Samples are along the x and y axes, and color intensi [file FEB4-13-617-s001.docx]

**Genomic and epigenetic regulation of HGSOC Subtypes**

In addition to understanding transcriptomic differences, we are also interested in identifying subtype-specific upstream genomic and epigenetic regulators. Since the subtypes were defined based on microRNA, methylation, and copy number variation (CNV) data in addition to mRNA expression, it follows that each subtype should also have distinct sets of differentially expressed microRNAs (DE-miRNAs), differentially methylated positions (DMPs), and genes with significant CNAs (**Supplemental Table S4**). To determine upstream genomic and epigenetic regulations of each subtype, we identified mRNAs significantly correlated with DE-miRNAs or with DMPs and examined the enrichment of these miRNA and methylation correlation signatures for subtype specific DEGs and key regulators (**Supplemental Tables S4 and S5**).

The impact of DE-miRNAs on gene expression varies by subtype. S-ECM and S-CC-2 have the largest number of DE-miRNAs compared to the other subtypes (**Supplemental Table S4**), and therefore gene signatures from these subtypes are likely more affected by miRNA regulation. In contrast, S-CC, S-LOC, and S-cillium each have fewer than 12 DE-miRNAs and consequently less miRNAs influence (**Supplemental Table S4**). We call the genes that are significantly correlated with DE-miRNAs DE-miRNA-affected genes, and **Supplemental Table S5** shows the enrichment of subtype-specific DE-miRNA-affected genes for subtype DEGs. Over 70% of upregulated DEGs and almost half of downregulated DEGs in S-ECM are significantly correlated with its DE-miRNAs, and about 40% of upregulated and 63% down-regulated S-CC-2 DEGs are correlated with DE-miRNAs. Additionally, many of the upregulated genes in S-immune are also likely under miRNA regulation (**Supplemental Tables S3 and S4**).

Enrichment for MSigDB gene sets in DE-miRNA-affected genes shows that miRNAs influence many subtype-specific processes. Downregulated DE-miRNA-affected genes in S-CC-2 and the upregulated DE-miRNA-affected genes in S-immune are enriched for immune system process and immune response related genes, while the downregulated DE-miRNA-affected genes in S-ECM are enriched for cell cycle genes (**Supplemental Figures S1A and S1B**). Other processes that are also under miRNA regulation include downregulation of cell cycle in S-ECM, S-cilium, and S-motif, downregulation of generic transcription in S-LOC, upregulation cell cycle in S-CC and S-CC-2, upregulation of biological adhesion in S-ECM, and upregulation of homophilic cell-cell adhesion in S-cilium (**Supplemental Figures S1A and S1B**). Interestingly, a subset of S-cilium DE-miRNA-affected genes are also predicted to be regulated by miR29, miR133, and miR518c families (**Supplemental Figures S1A and S1B**). Finally, DE-miRNA-affected genes have high overlap with subtype key regulators, suggesting that miRNA-regulated genes function further upstream to drive downstream subtype-specific molecular processes (**Supplemental Table S5**).

We performed similar analyses on subtype-specific DMPs and discovered distinct methylation patterns in each subtype. Similar to subtype DE-miRNAs, S-ECM and the S-CC-2 also have the largest number of subtype-specific DMPs (**Supplemental Table S4**). To identify genes that are potentially regulated by methylation, we performed Spearman correlation analysis of gene expression and DMP M-values. Genes that are significantly negatively correlated with DMPs and also located near DMP sites are classified as cis-eDMP genes, and genes significantly correlated with DMPs regardless of their proximity to methylation sites are trans-eDMP genes. Cis-eDMP genes are primarily involved in upregulating immune system process in S-immune and the S-ECM (upregulated immune response) and downregulating immune system process in S-CC-2 (**Supplemental Figures S1C and S1D**). Genes containing conserved sequences for transcription factor binding, such as *E12, SP1, MAZ* and *AREB6*, are also predicted to be under cis-regulation by the subtype-specific DMPs in S-immune, S-ECM, S-CC-2, and S-cilium (**Supplemental Figure S1C and S1D**). Cis-eDMP genes also include a large portion of subtype-specific key regulators, especially those that are predicted to downregulate DEGs in the S-cilium and S-motif (**Supplemental Table S6**). Trans-eDMP genes are associated with upregulation of cell cycle signal in S-CC-2, as well as downregulation of ECM genes in S-immune (**Supplemental Figures S1E and S1F**). Additionally, the upregulated genes in S-CC and the downregulated genes in S-ECM are related to morphogenesis and organization (**Supplemental Figures S1E and S1F)**.


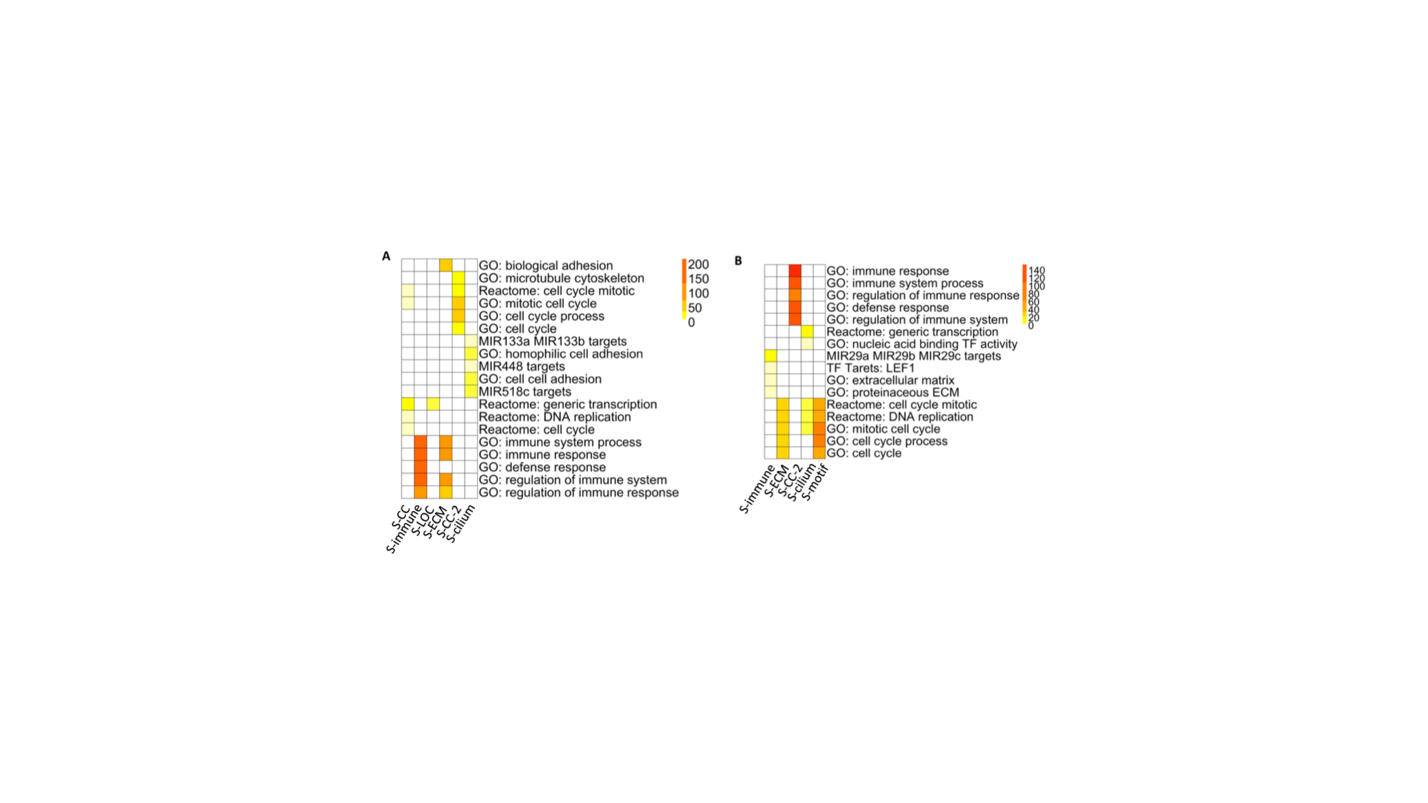


**
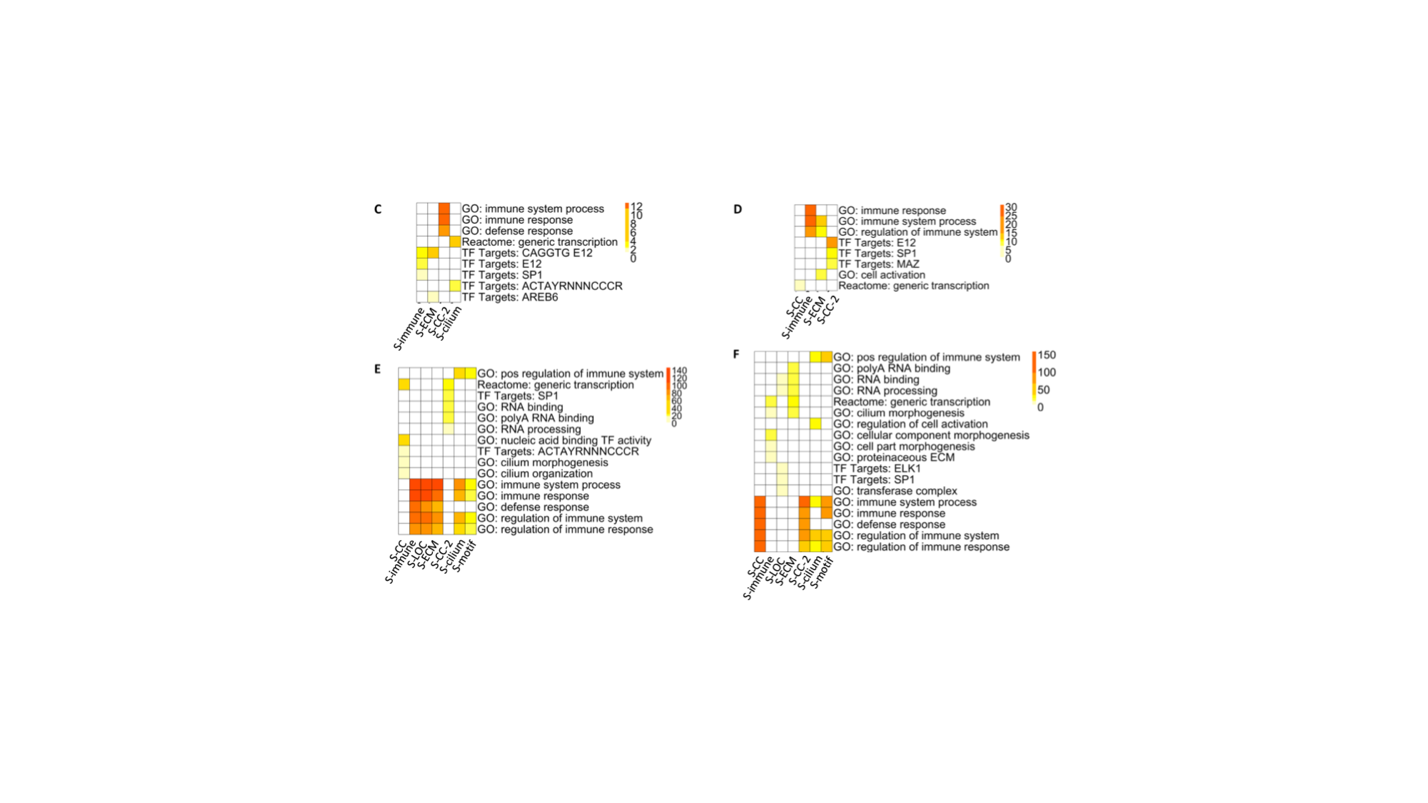
**

**
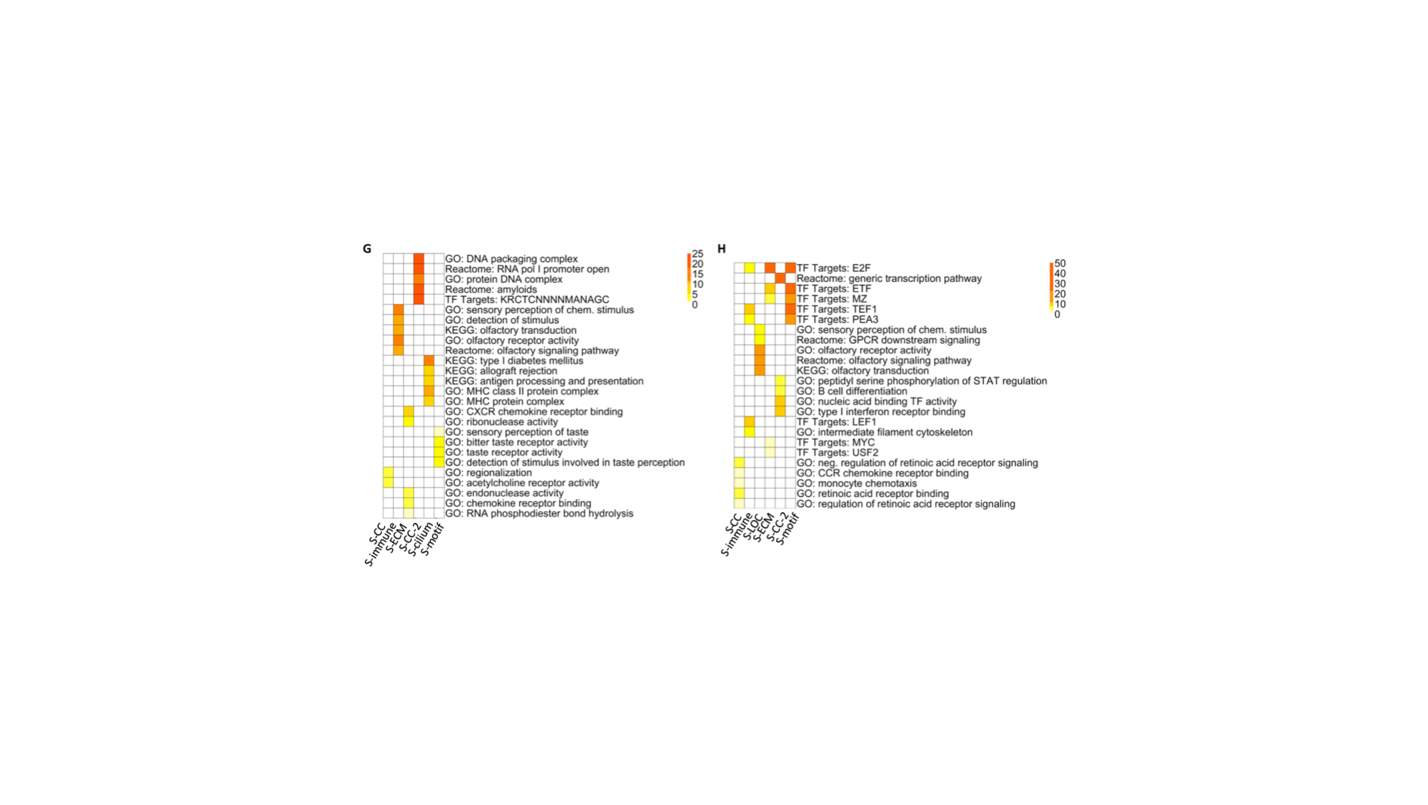
**

**
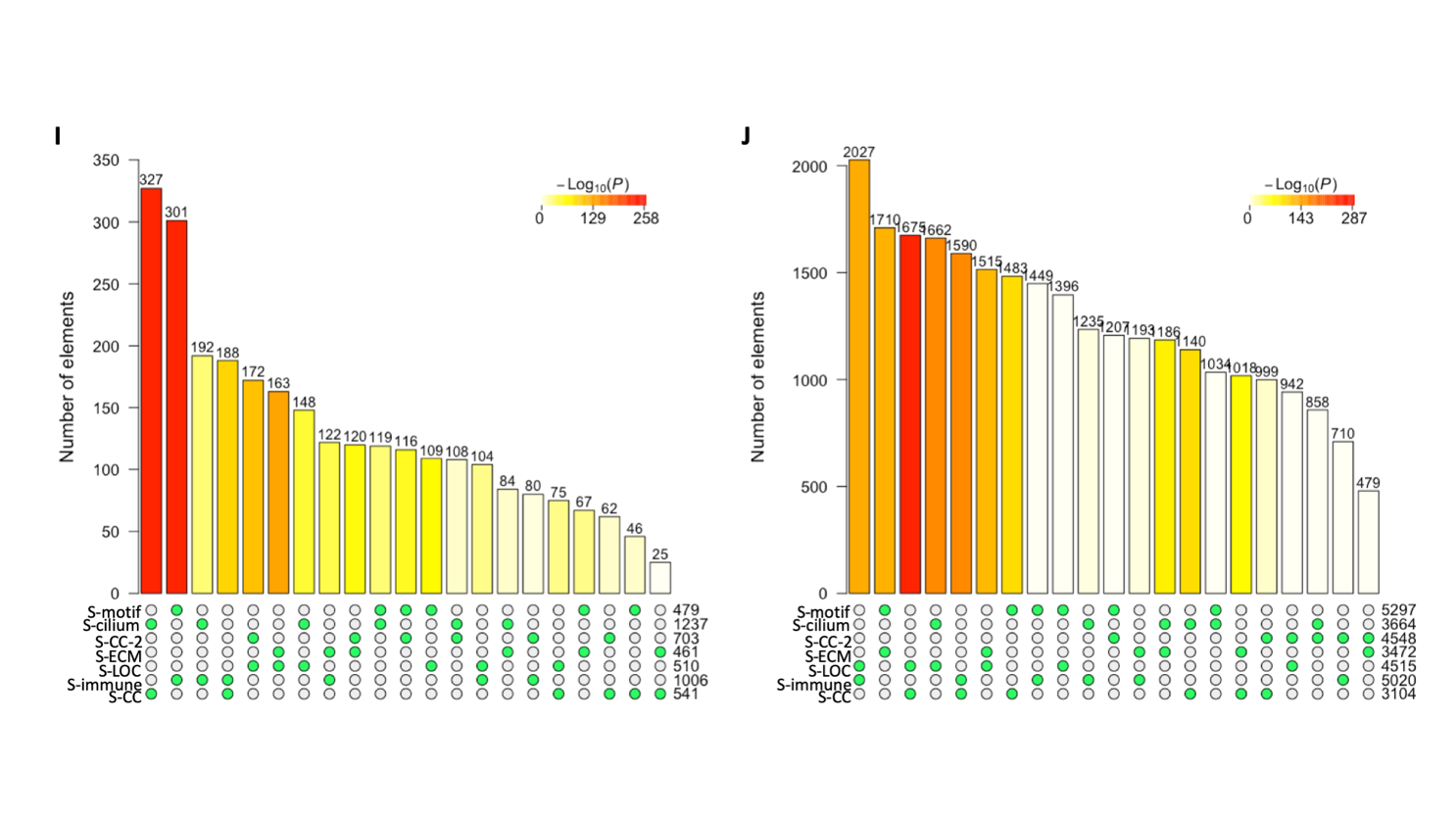
**

**Supplemental Figure S1. Functional annotation of upstream regulator signatures of the HGSOC subtypes. (A-B)** Heatmaps showing enrichment of MSigDB gene sets in (A) upregulated genes and (B) downregulated genes for each set of subtype DE-miRNA-affected genes. DE-miRNAs were identified by comparing the samples in a subtype with the rest of the samples.  **(C-D)** Heatmaps showing enrichment of MSigDB gene sets in the subtype cis-eDMP genes for (C) hypermethylated and (D) hypomethylated DMPs. **(E-F)** Heatmaps showing enrichment of MSigDB gene sets in genes (E) upregulated and (F) downregulated for each subtype that are also predicted to be under trans-regulation by subtype DMPs. DMPs were identified by comparing the samples in each subtype with the rest of the samples.  **(G-H)** Heatmaps showing enrichment of MSigDB gene sets in the predicted (G) amplified and (H) deleted genes for each subtype. **(I-J)** Bar plots showing number of overlapping (I) amplified and (J) deleted genes between subtype pairs. Green dots below each bar indicate the subtype pairs being compared.

Enrichment test is done by FET. Color gradient shows -log_10_(FET Bonferroni-corrected p-value).

Finally, we identified subtype-specific CNAs using GISTIC2.0 [35], which produced different patterns of genomic locations with copy number variations for each subtype and predicted many more deletions than amplifications for all the subtypes (**Supplemental Figures S6 and S7; Supplemental Table S4**). Consequently, there is a larger proportion of downregulated DEGs and associated key regulators with CNVs compared to upregulated DEGs (**Supplemental Table S7**). The amplified genes in S-immune are associated with sensory perception and G-protein coupled receptor (GPCR) related pathways while amplifications in S-cilium are involved in autoimmune disease and MHC (**Supplemental Figure S1G**). Deletions in S-CC and S-immune are related to retinoic acid regulation and signaling, and deletions in S-LOC are mainly involved in GPCR signaling (**Supplemental Figure S1H**). Amplifications and deletions in S-CC-2 are involved in DNA packaging/chromatin assembly and immune activation, respectively, which is consistent with the previous analysis of DE-miRNA and DMP correlated genes in the subtype (**Supplemental Figures S1G and S1H**), and likely supports the upregulated cell cycle processes that characterize S-CC-2. Most of the common gene amplifications and deletions involved in HGSOC tumorigenesis are simultaneously present in most subtypes and do not show subtype-specific differences, except for *AKT1/2* amplifications and *BRCA1/2* deletions present in only two of the seven subtypes (**Supplemental Table S8**). Lastly, there is little overlap in both amplified and deleted genes within subtype pairs with poor prognoses relative to their total CNAs. S-ECM and S-CC-2 have 120 overlapping amplified genes and 479 overlapping deleted genes, which makes up 17%-26% of amplified and 10%-14% of deleted genes, respectively, for these subtypes (**Supplemental Figure S1I and S1J**).

**
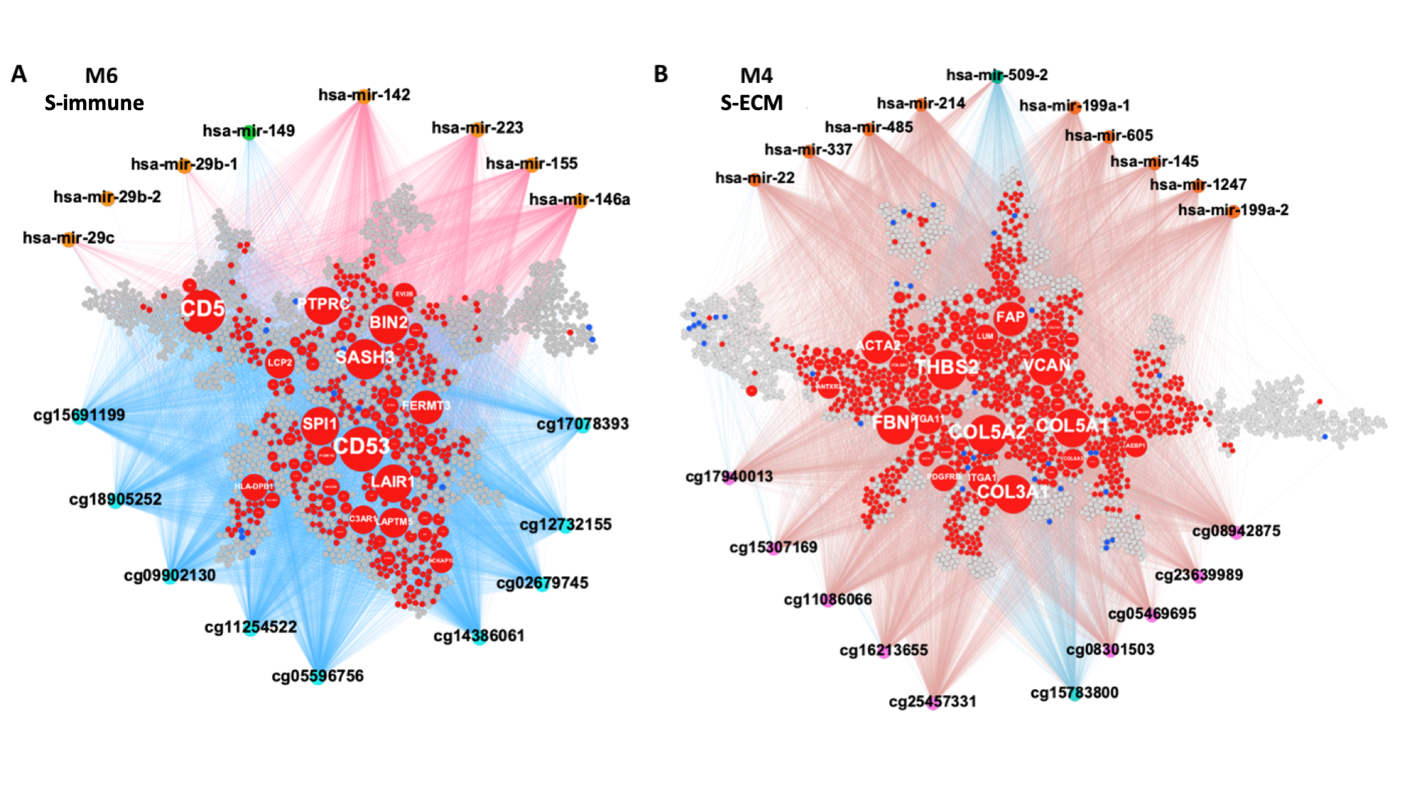
**

**
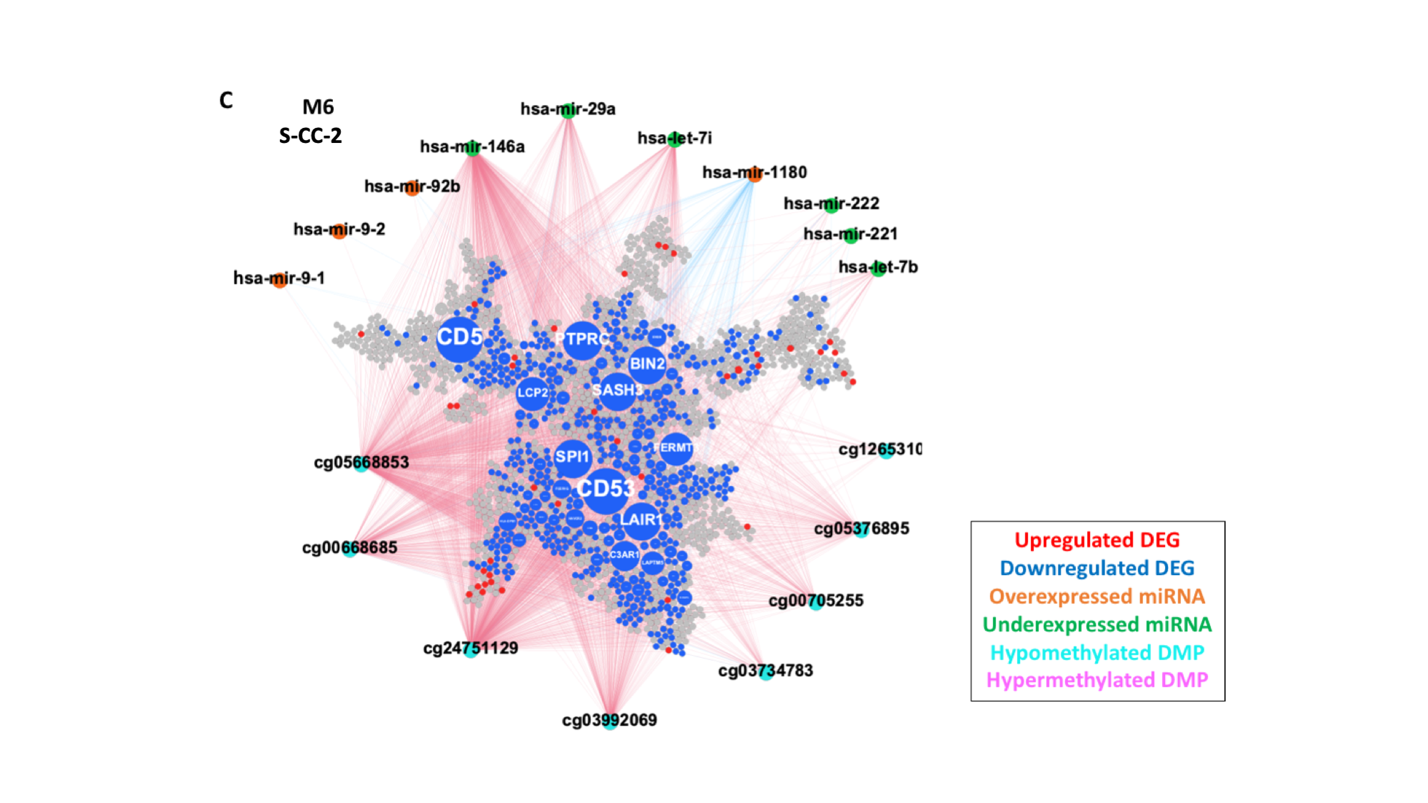
**

**Supplemental Figure S2. Subtype specific DE-miRNAs and DMPs and their relationships with MEGENA network modules**. Graphical representation of regulation of subtype-specific DEGs by top 10 subtype DE-miRNAs and DMPs. DEGs are highlighted by color within MEGENA network modules, and DEGs labeled in white are also subtype key regulators. DE-miRNAs and DMPs are also color coded as shown. Edges between MEGENA module nodes and DE-miRNAs and DMPs show significant positive (red) or negative (blue) expression correlations. **(A)** S-immune DE-miRNAs and DMPs and the immune system process-enriched module M6. **(B)** S-ECM DE-miRNAs and DMPs and the ECM-enriched module M4. **(C)** S-CC-2 DE-miRNAs and DMPs and the immune system process-enriched module M6.

Modules enriched for the subtype-specific DEG signatures are highly affected by upstream genomic and epigenetic regulation, though the degree of regulation varies by subtype. miRNAs exert a larger degree of regulation on modules M4 and M6 with respect to S-immune, S-ECM, and S-CC-2 (**Supplemental Figure S2**). The majority of genes in module M6 that are upregulated in S-immune are significantly correlated with just a handful of miRNAs (**Supplemental Figure S2A**). For example, miR-142 is a tumor suppressor in a variety of cancers such as lung and breast, and it is likely a major upstream regulator of the increased immune activation signature observed in the S-immune [36-38]. In addition, miR-155, which is upregulated in S-immune but downregulated in S-CC-2, has been shown to be a key regulator of immune response in the context of anti-tumor immunity (**Supplemental Figures S2A and S2C**) [39].


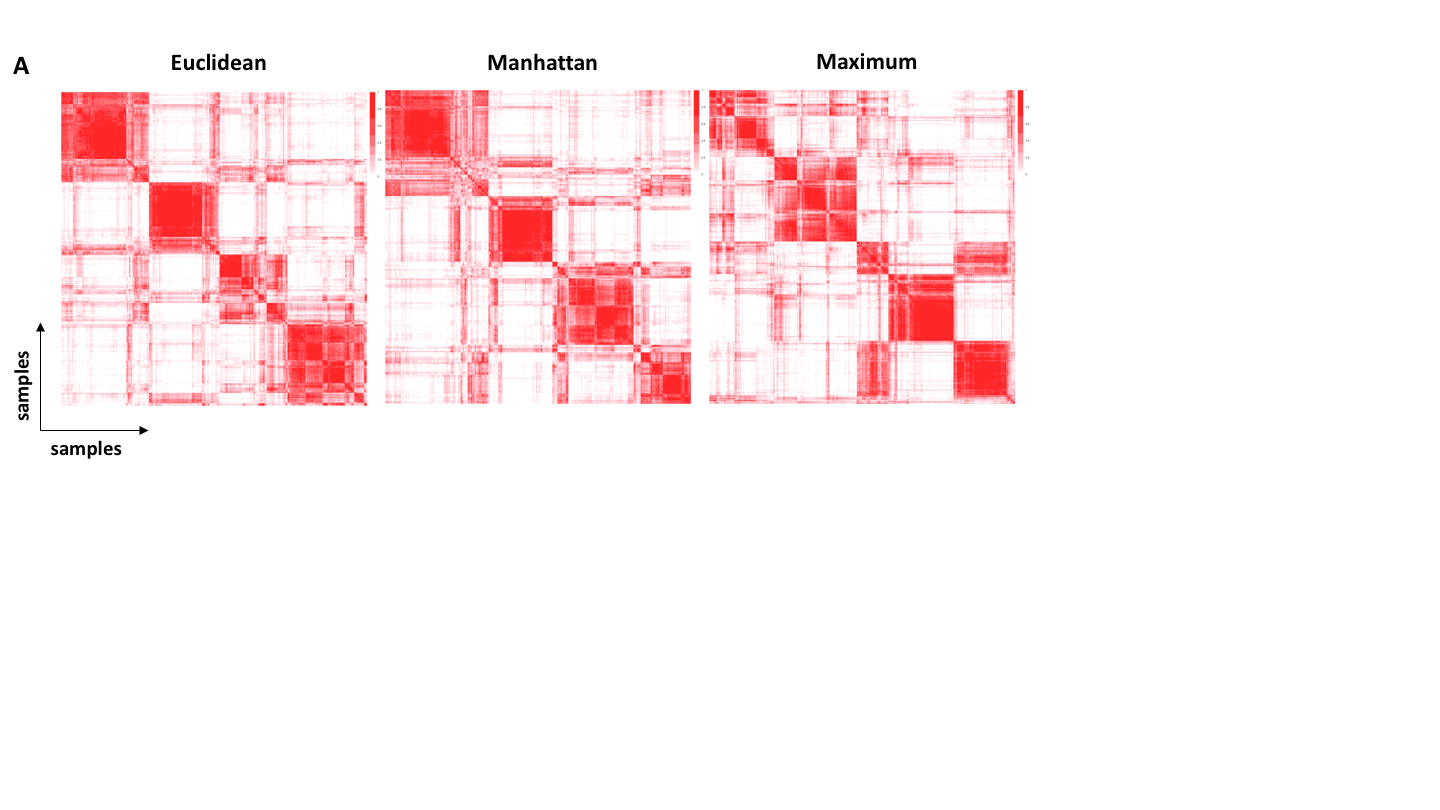


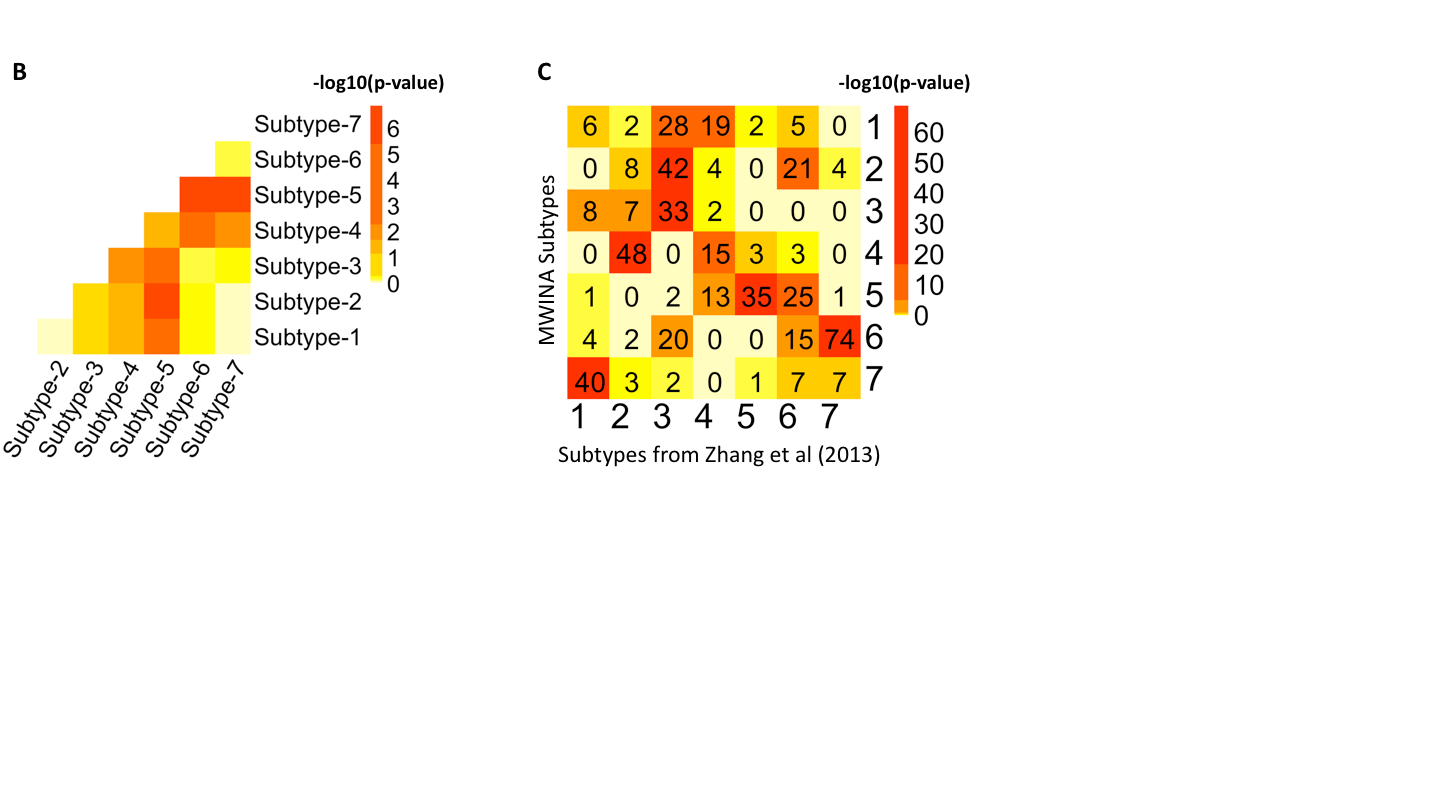


**Supplemental Figure S3. MWINA subtypes are robust redistributions of Zhang et al HGSOC subtypes, and have some differences in age at diagnoses. (A)** Plots of HGSOC sample concordances. Samples are along the x and y axes, and color intensities correspond to sample pair-wise concordance probabilities. Concordance probabilities range from 1.0 along the diagonal (red) to 0 (white). Concordance clustering is based on Euclidean, Manhattan, or Maximum distance calculation methods.

**(B)** Heatmap of -log_10_(p-value) from Student’s t-test for age at diagnosis between subtype pairs. Only comparisons between Group 4 or Group 5 versus other subtypes were significant (p < 0.05).

**(C)** Heatmap of -log_10_(p-value) from Fisher’s Exact Test for overlap between patients from MWINA subtypes and patients from Zhang et al (2013) subtypes.


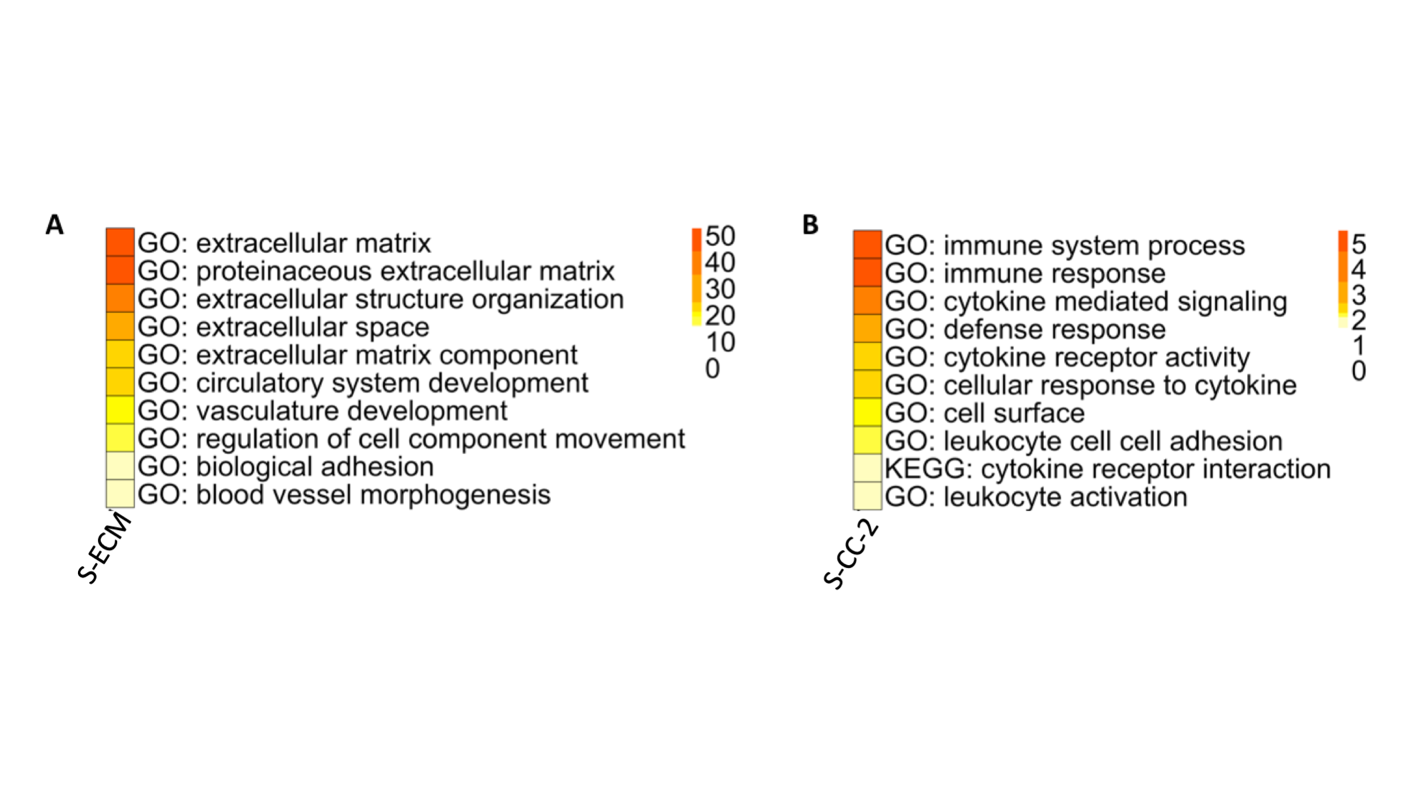


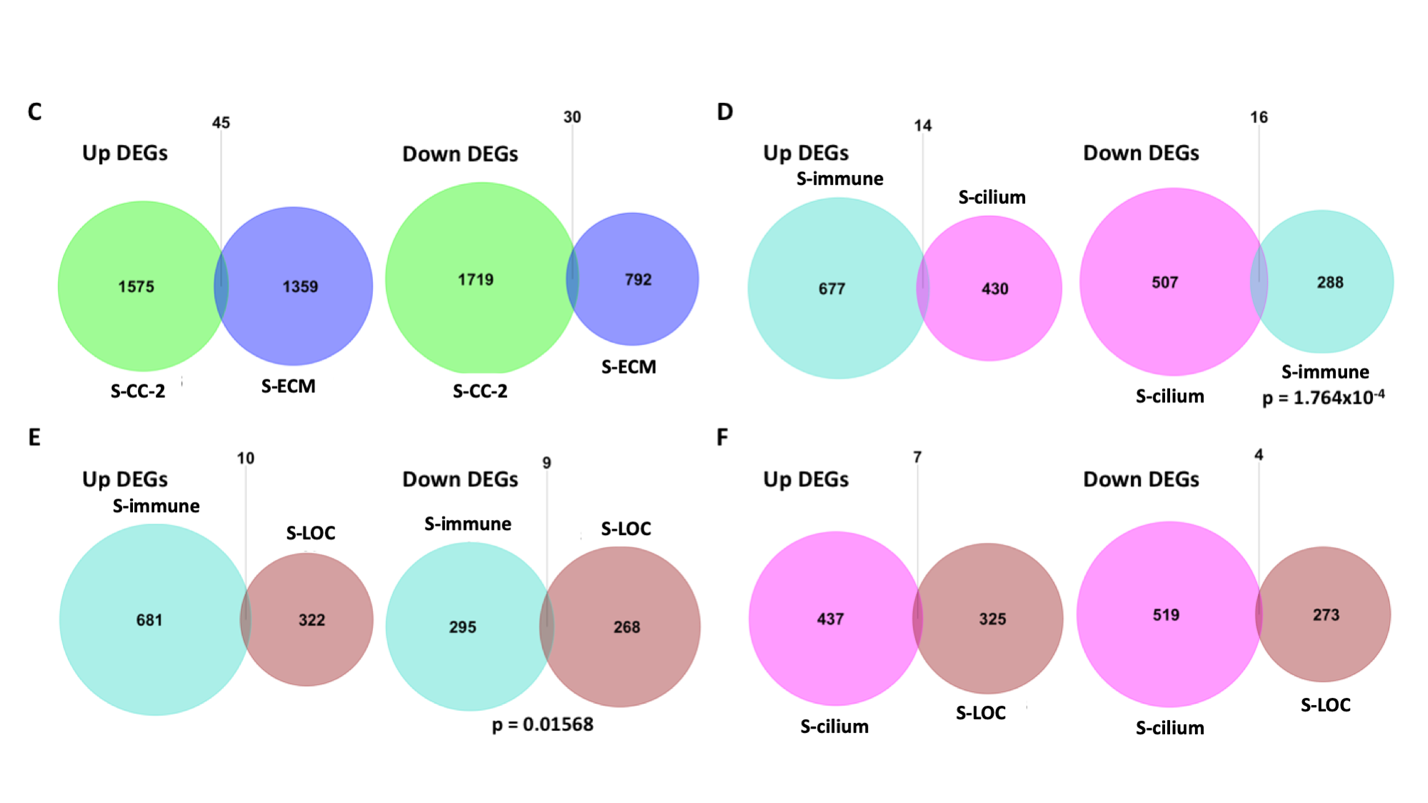


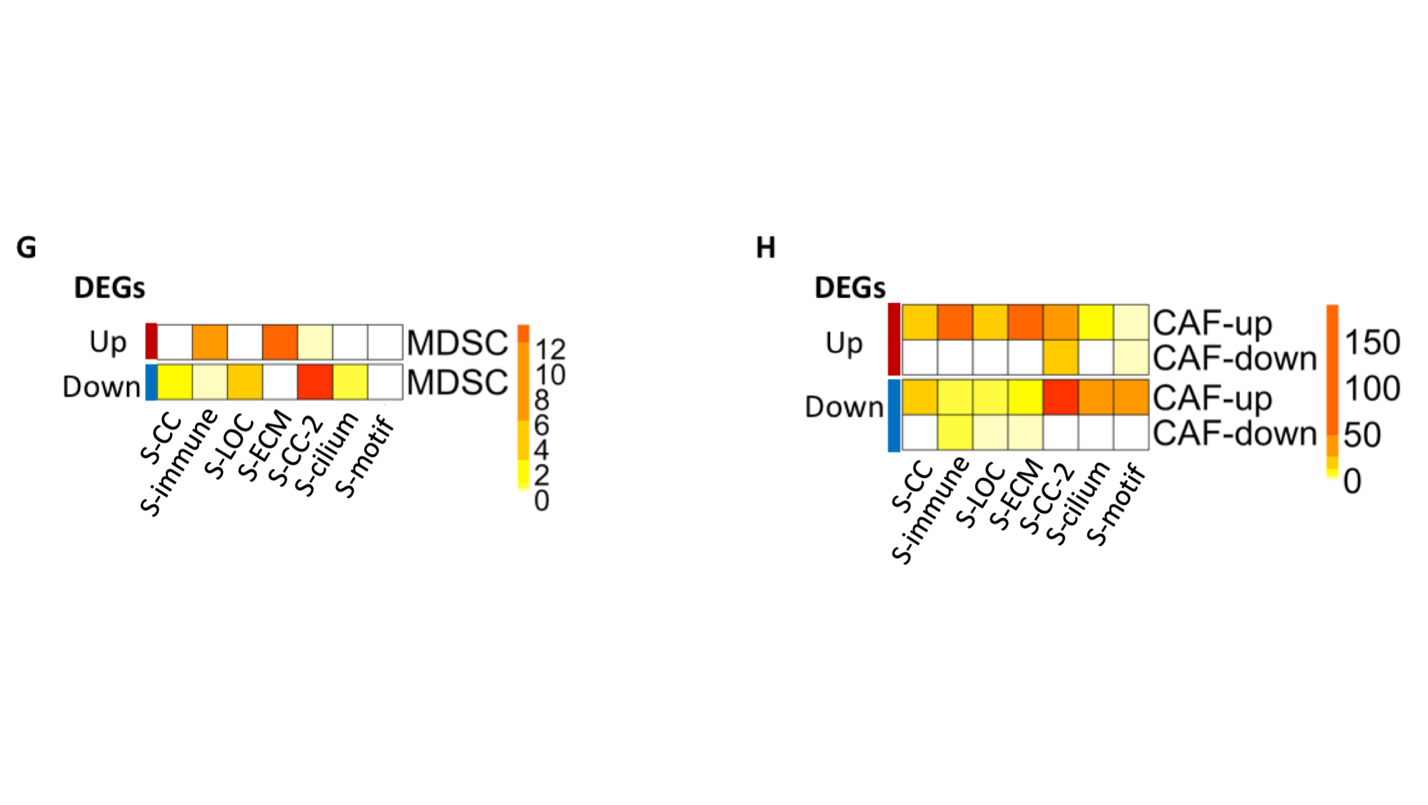


**Supplemental Figure S4. HGSOC subtypes with similar prognosis have distinct gene expression signatures. (A-B)** Heatmap of MSigDB gene set enrichment for overexpressed (A) and underexpressed (B) genes in HGSOC tumor samples of each subtype with respect to normal controls. Enrichment is represented by FET -log_10_(BH adjusted p-value) for gene set overlap.

**(C-F)** Overlap of upregulated or downregulated DEGs between (C) poor surviving subtypes (S-ECM and S-CC-2) and (D-F) favorable survival subtypes (S-immune, S-LOC, and S-cilium). P-value is calculated from Fisher’s Exact Test (FET), and non-significant FET p-values (p > 0.05) are not shown.

**(G-H)** Heatmaps showing subtype DEG enrichment for genes up- or downregulated in (D) CAFs from ovarian tumors compared to normal ovarian stroma, and for genes upregulated in (E) MDSCs (compared to PMNs) from NSCLC. Enrichment is determined via FET. Color gradient shows -log_10_(FET p-value).

FET, Fisher’s Exact Test; CAF, cancer associated fibroblast; MDSC, myeloid derived suppressor cell; NSCLC, non-small cell lung cancer

Related to **Figure 2.**


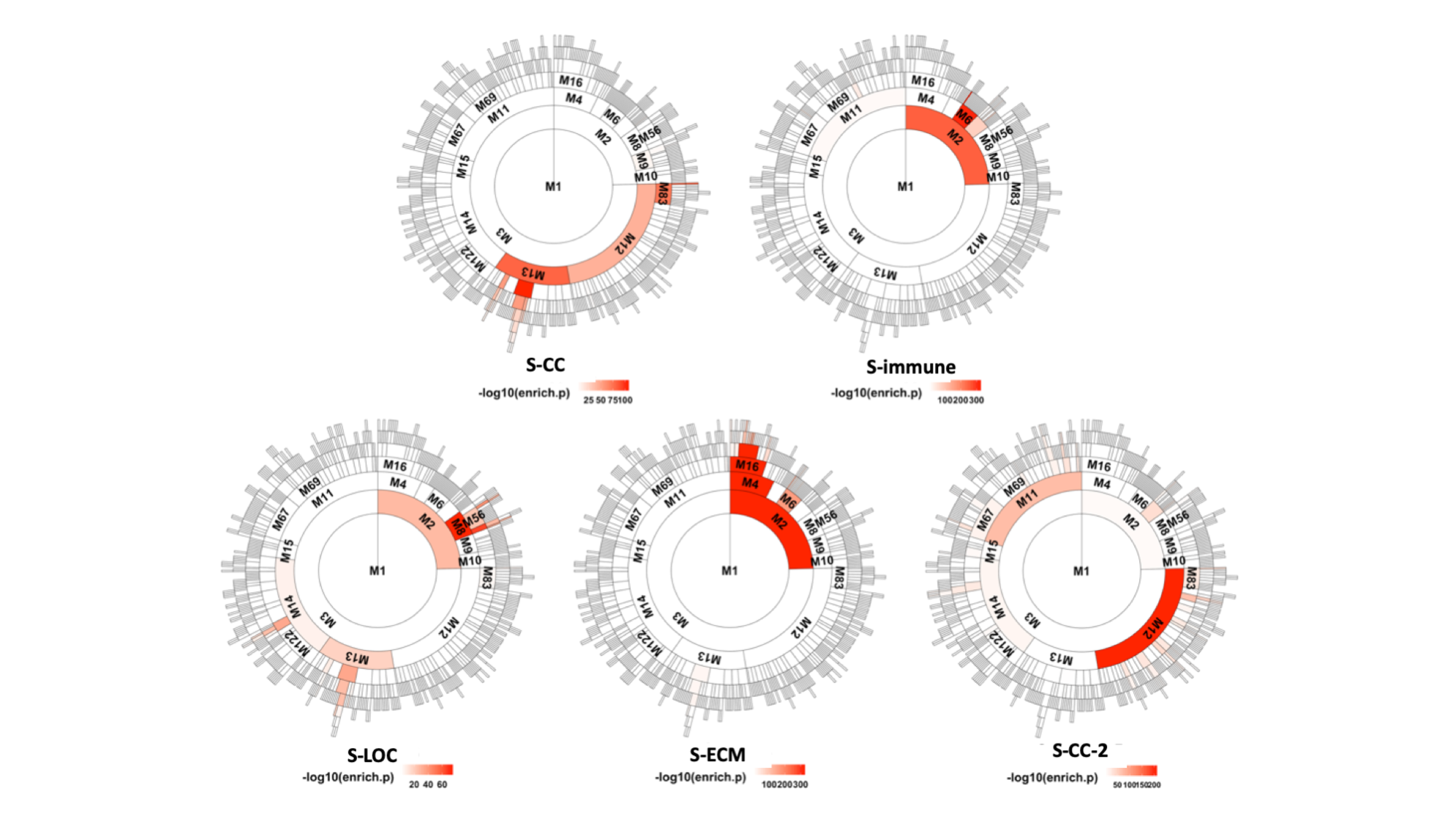


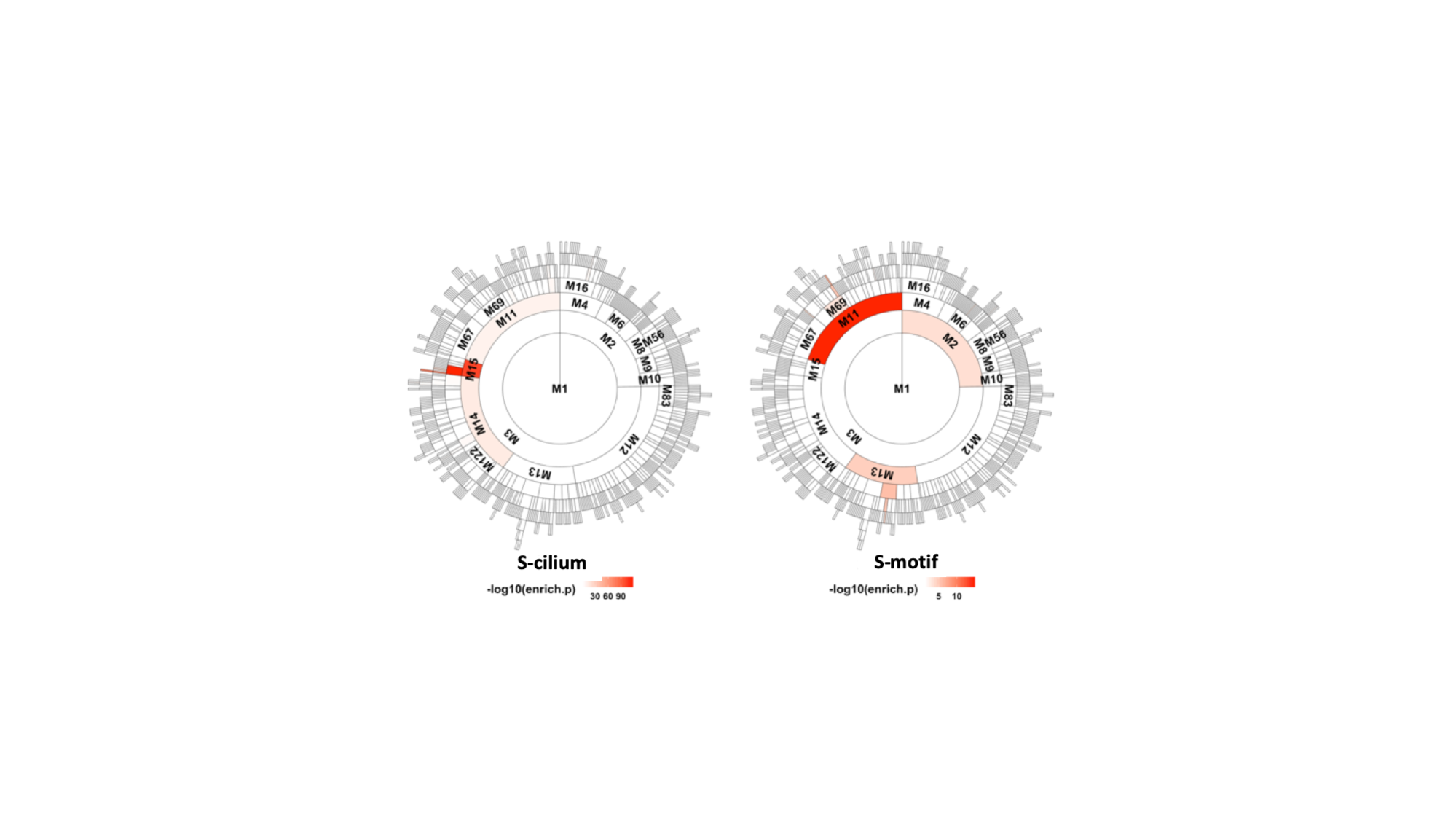


**Supplemental Figure S5. MEGENA module genes are enriched for HGSOC subtype upregulated DEGs.** Sunburst plots showing overlap of subtype-specific upregulated DEGs with MEGENA network module genes. Colors correspond to -log_10_(FET p-value). Modules with p-value > 0.05 are not colored.

Related to **Figure 3**.


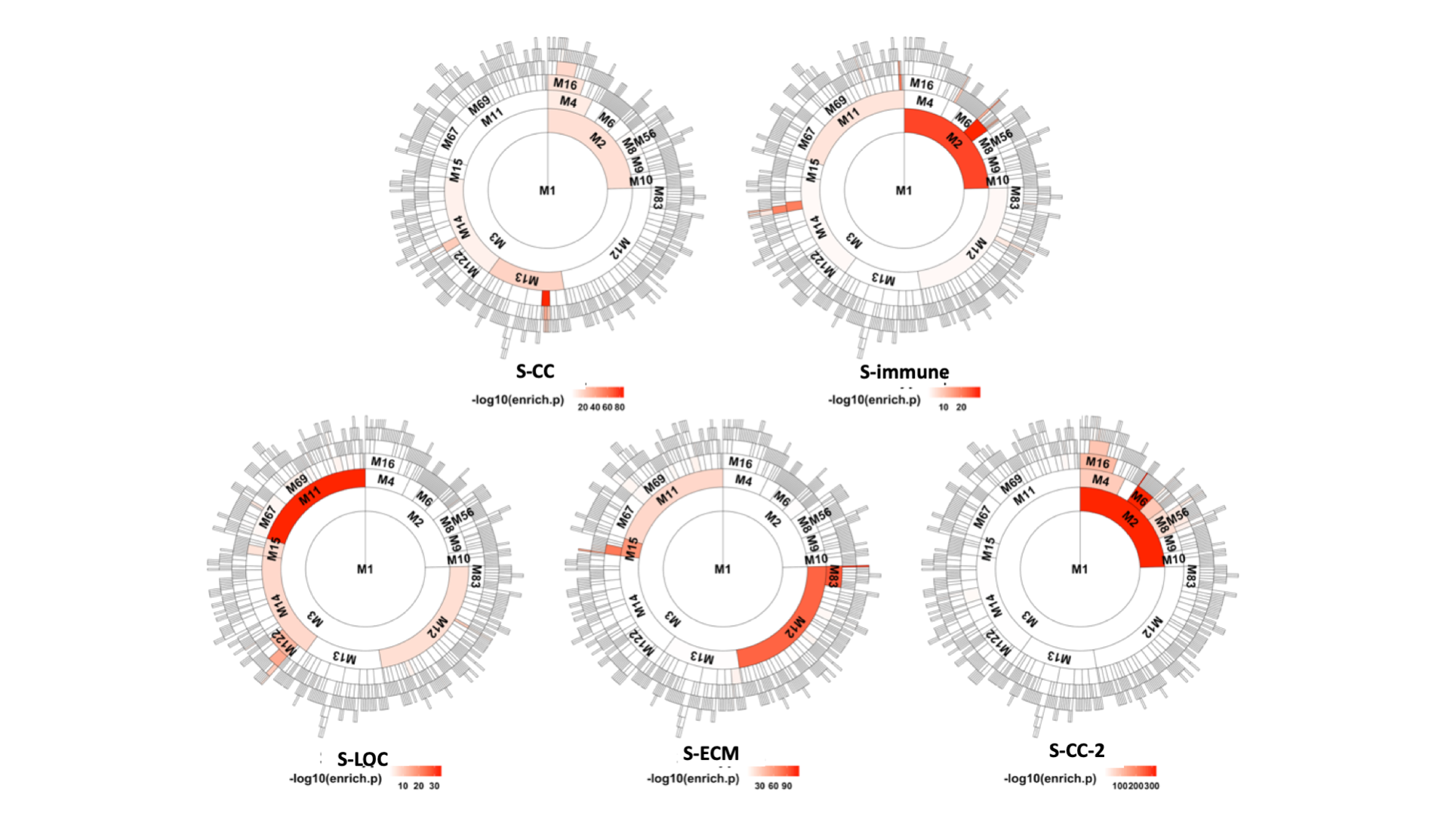


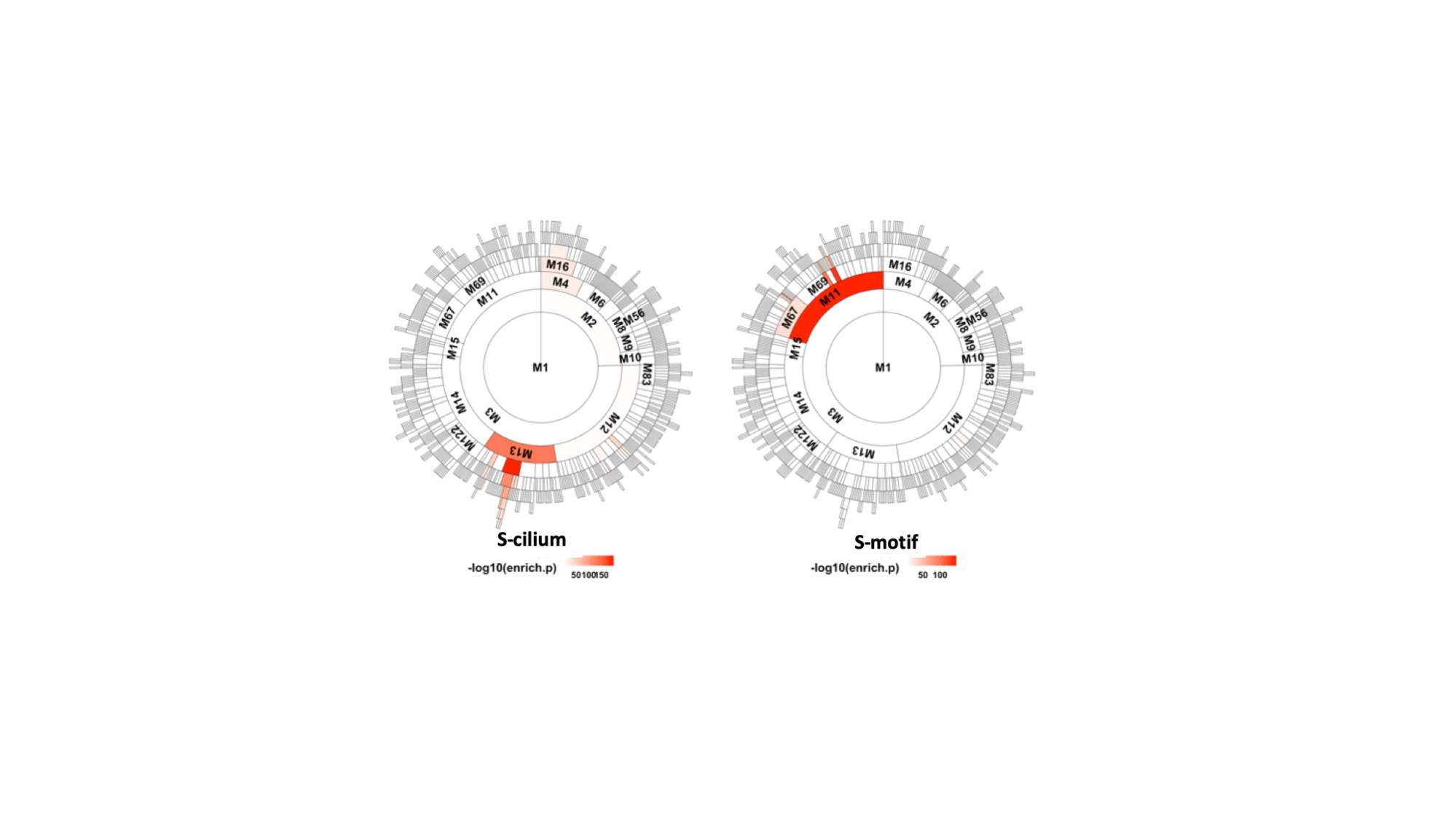


**Supplemental Figure S6. MEGENA module genes are enriched for HGSOC subtype downregulated DEGs.** Sunburst plots showing overlap of subtype-specific downregulated DEGs with MEGENA network module genes. Colors correspond to -log_10_(FET p-value). Modules with p-value > 0.05 are not colored. Related to **Figure 3**.

**
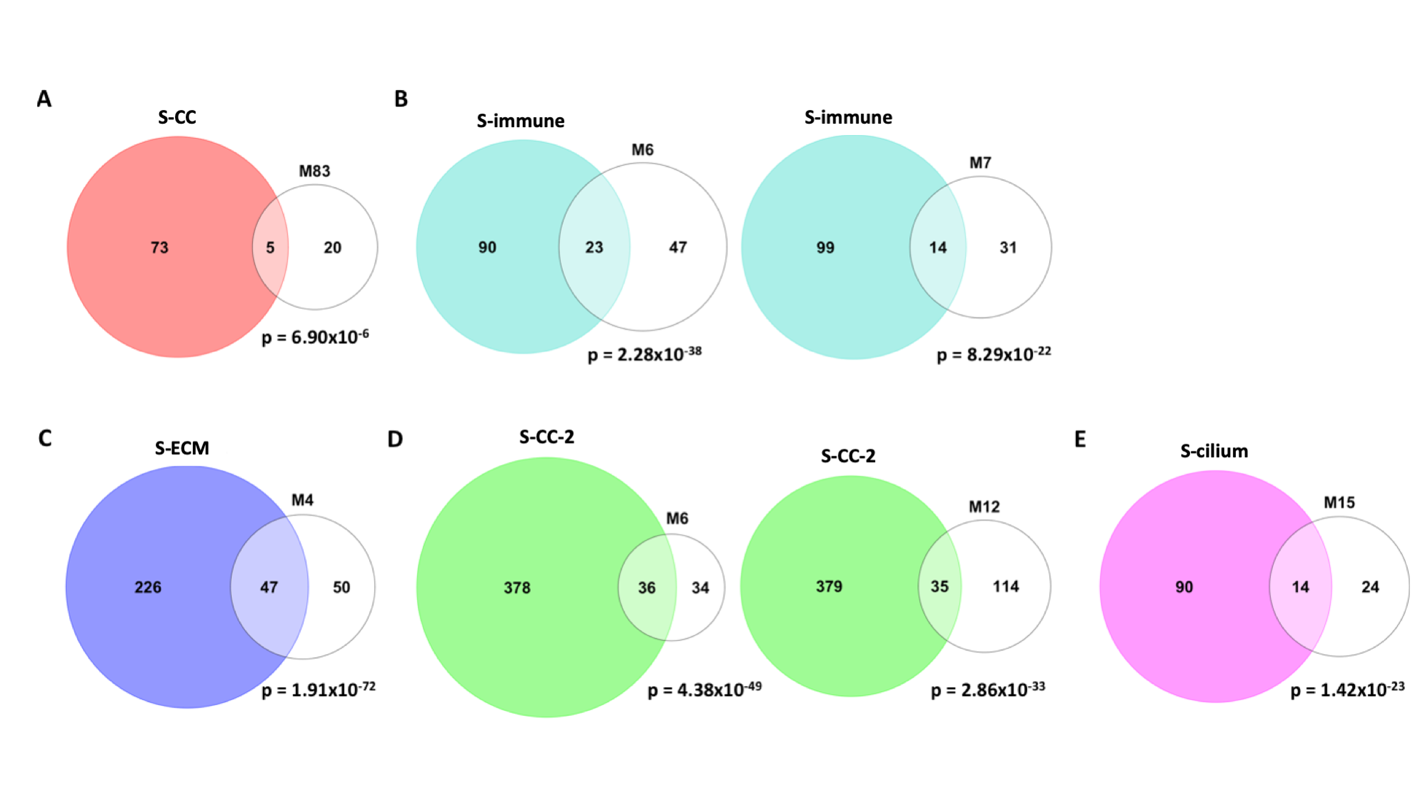
**

**
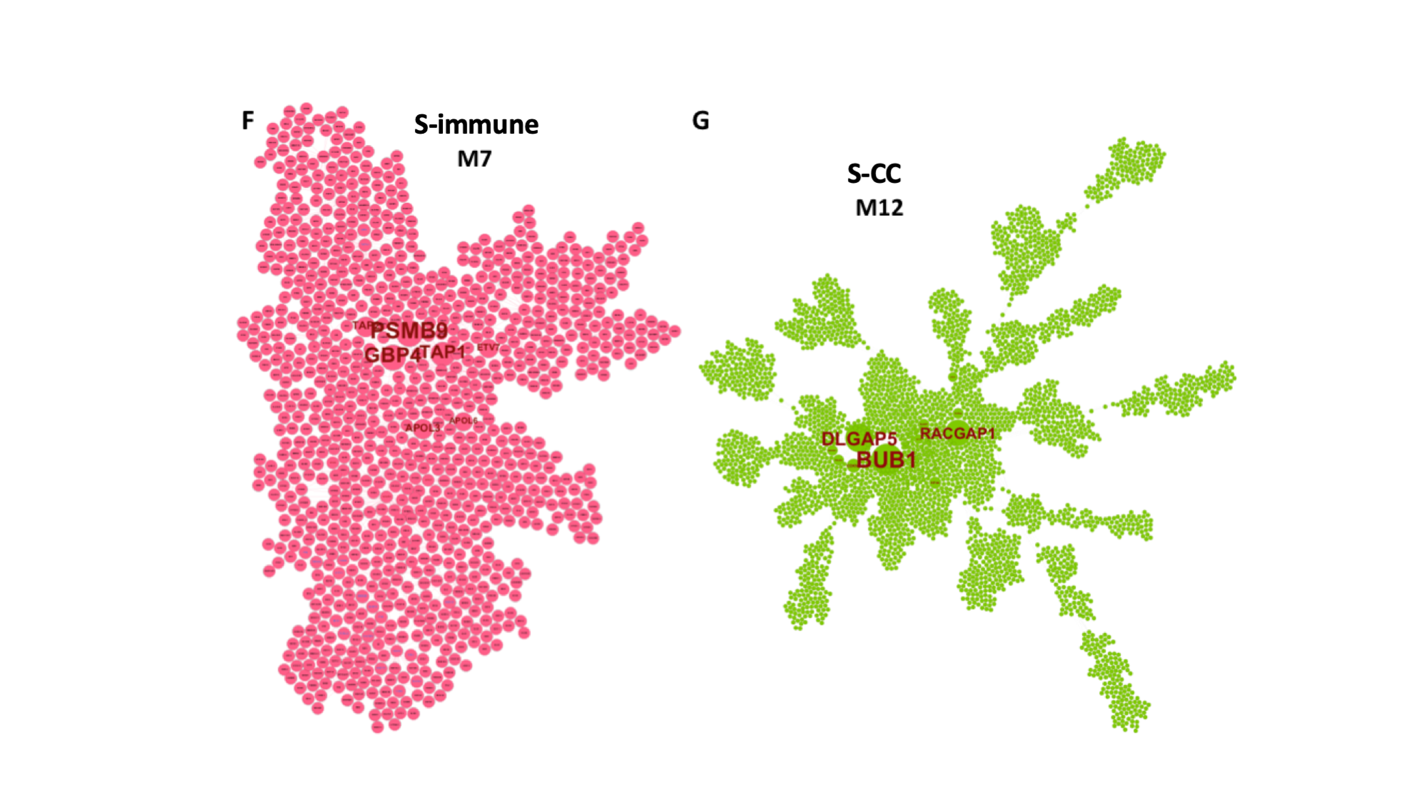
**

**Supplemental Figure S7. Overlap of subtype-specific key driver genes and MEGENA module genes. (A-E)** Overlap of subtype-specific key driver genes and MEGENA module hub genes for (A) S-CC and cell cycle-enriched module M83, (B) S-immune and immune system process-enriched module M6 and interferon response-enriched module M7, (C) S-ECM and ECM-enriched module M4, (D) S-CC-2 and immune system process-enriched module M6 and cell cycle-enriched module M12, and (E) S-cilium and cilium-enriched module M15. P-value is calculated from Fisher’s Exact Test (FET)

ECM, extracellular matrix; FET, Fisher’s Exact Test. **(F-G)** Subtype-specific key drivers shown in MEGENA modules. Node size and label size are proportional to key driver ranking, with larger node and text sizes corresponding to higher ranked key drivers. Label color represents direction of subtype-specific DEGs (p < 0.05) used for key driver prediction, where red corresponds to upregulated DEGs and blue corresponds to downregulated DEGs. (F) S-immune key drivers shown in the interferon response-enriched module M7. (G) S-CC key drivers in the cell cycle-enriched module M12. Related to **Figure 4**.

**
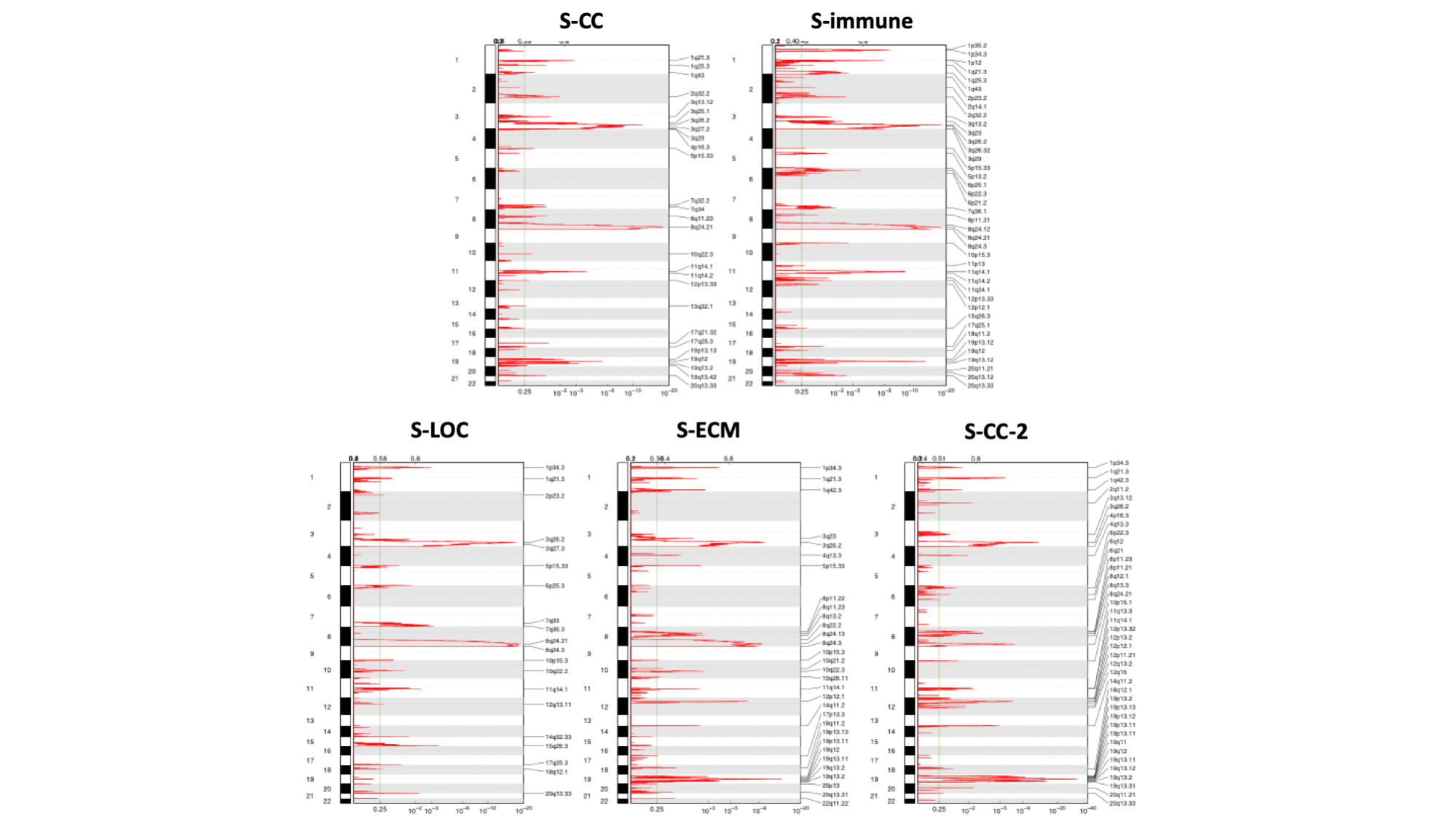
**

**
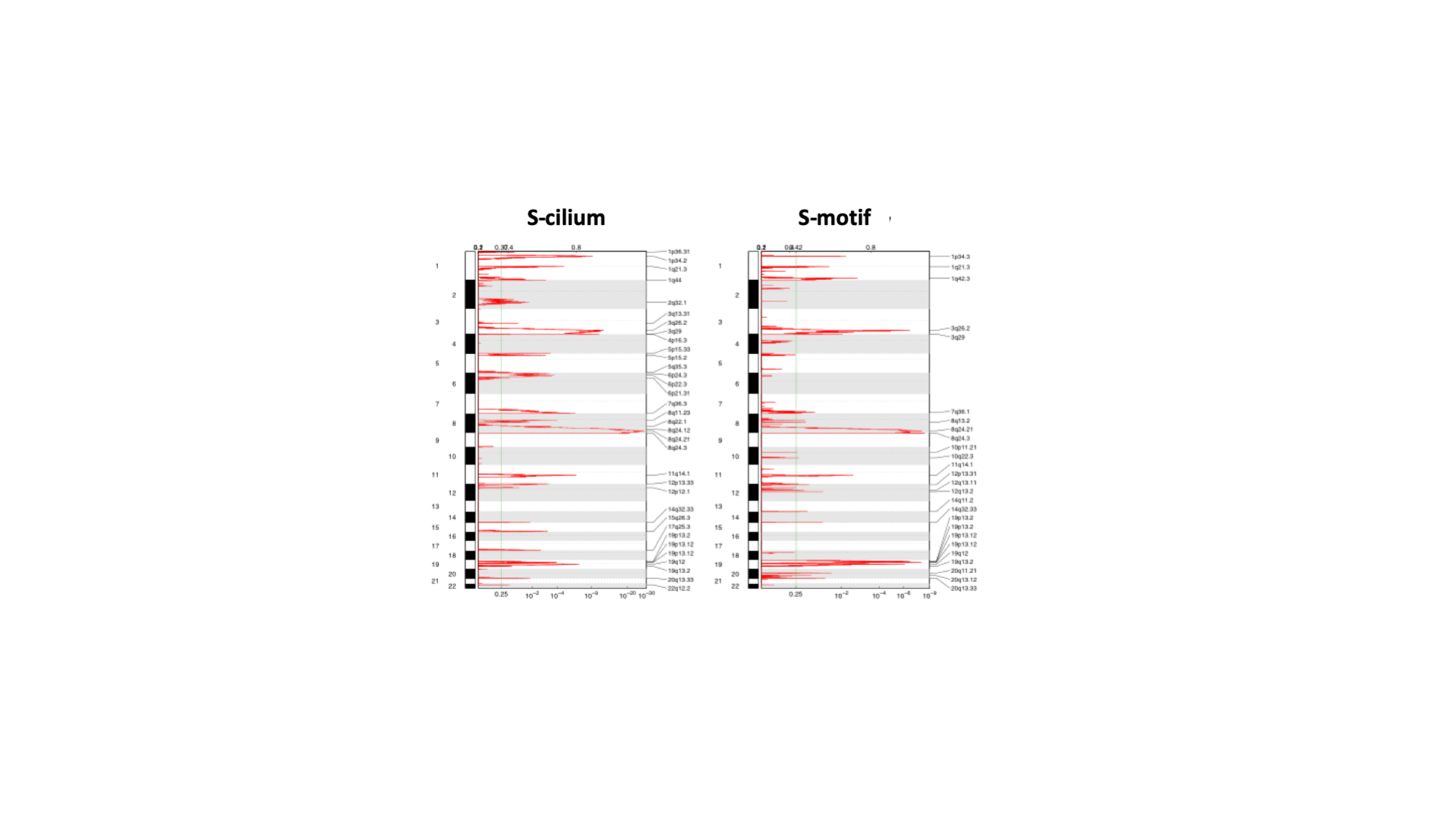
**

**Supplemental Figure S8.** Focal somatic copy number alterations for subtype samples by chromosome location of amplified regions.


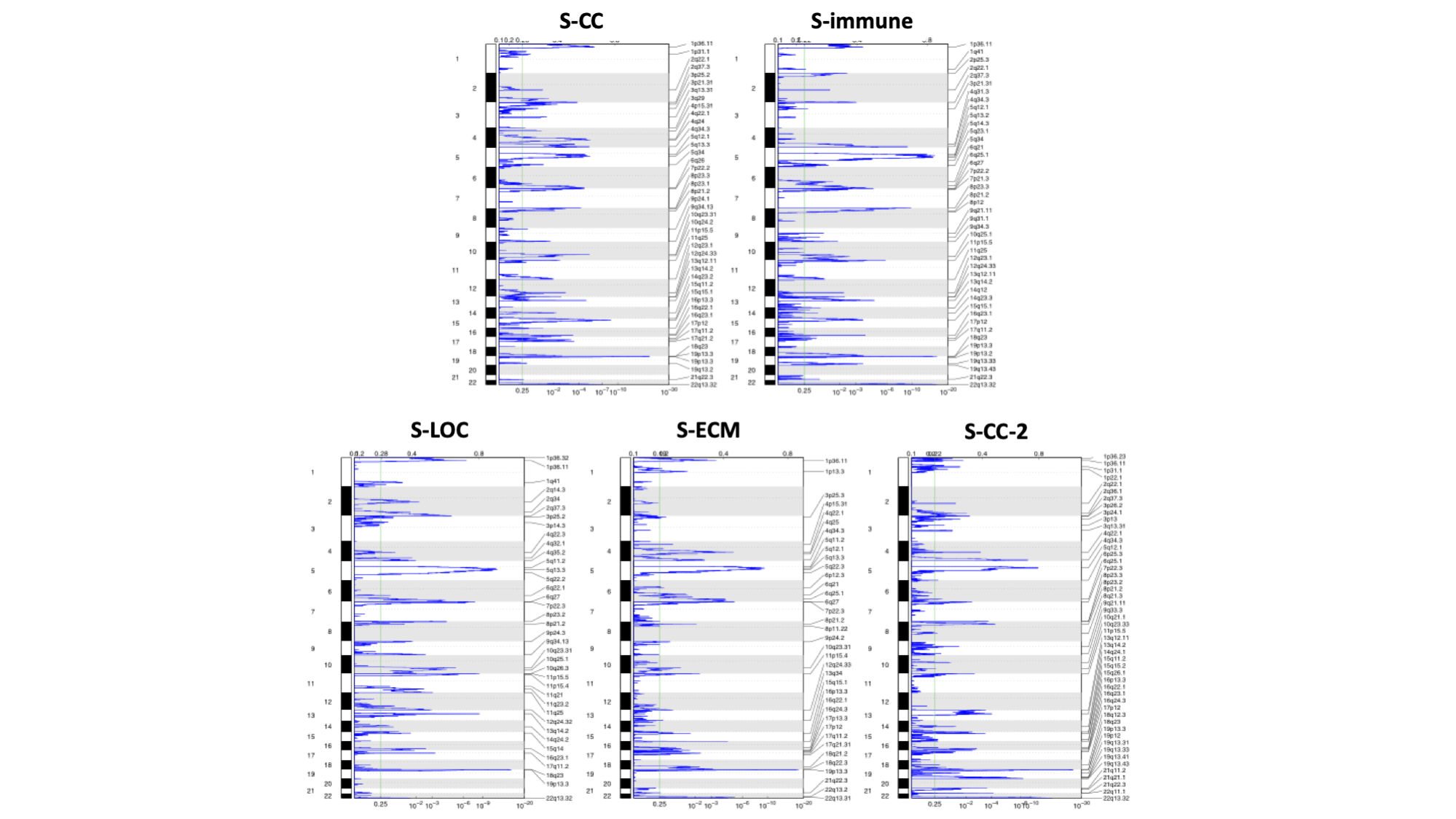


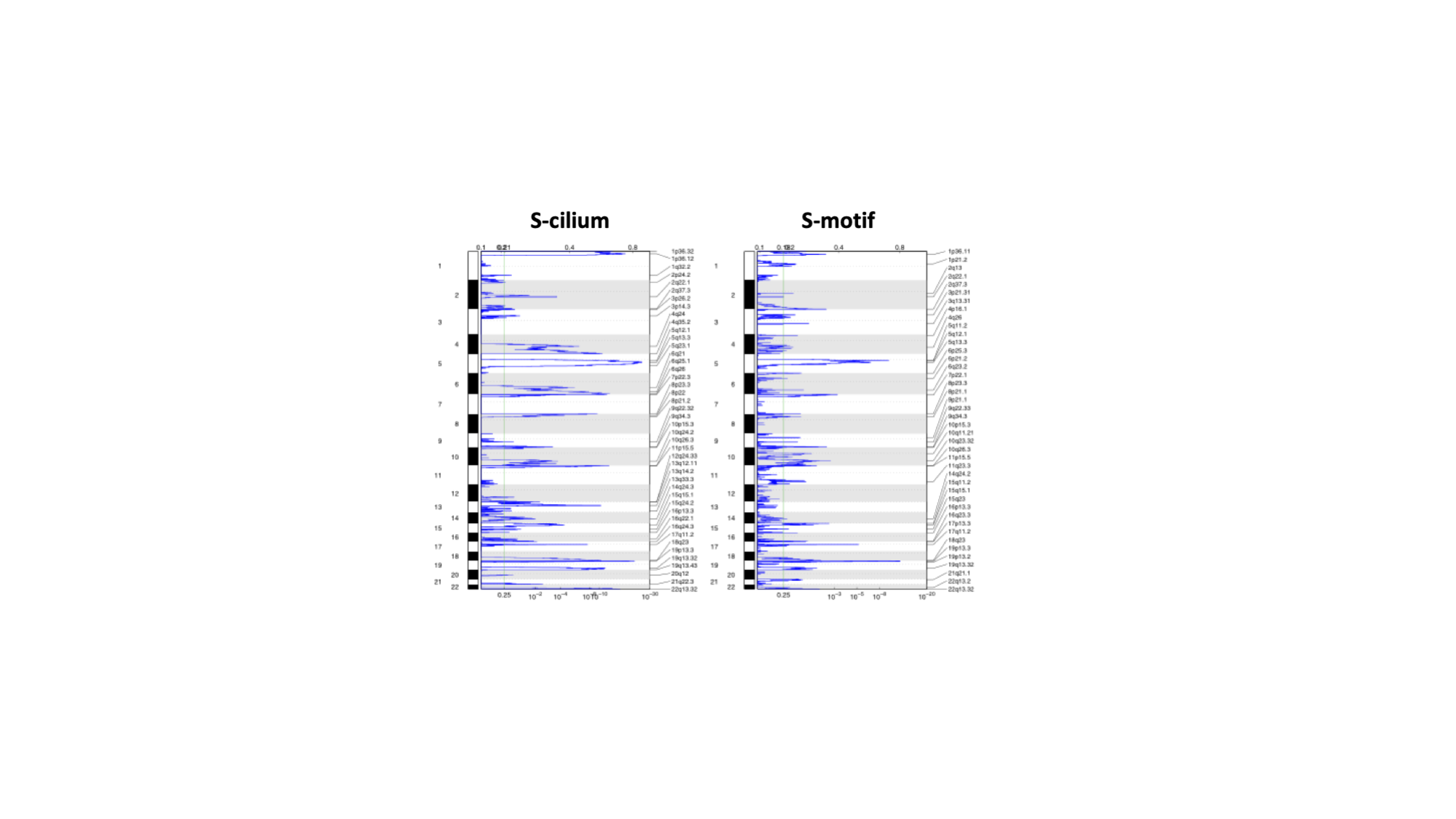


**Figure S9.** Focal somatic copy number alterations for subtype samples by chromosomal location for deleted regions.

**Supplemental Table S1. Kruskal-Wallis test for significance of clinical traits and ESTIMATE scores in subtypes**

| **Trait** | **KW p-value** |
| --- | --- |
| Age at Diagnosis | 2.906E-07 |
| Age at Diagnosis (Subtype-4 excluded) | 1.06E-06 |
| Age at Diagnosis (Subtype-5 excluded) | 0.03411 |
| Age at Diagnosis (Subtype-4 & 5 excluded) | 0.8124 |
| Stage | 0.2493 |
| Hist. Grade | 0.1429 |
| Residual Tumor | 6.76E-02 |
| ESTIMATE Stromal | 1.46E-20 |
| ESTIMATE Immune | 1.22E-17 |
| ESTIMATE Tumor Purity | 1.46E-20 |

**Supplemental Table S2. Student t-test of clinical traits between samples with RNA-Seq data and samples used for subtype identification**

| **Clinical Trait** | **mRNA T-test**  **p-value** | **mRNA T Statistic** | **miRNA T-test**  **p-value** | **miRNA T Statistic** |
| --- | --- | --- | --- | --- |
| Age at Diagnosis | 0.7255 | 0.35126 | 0.9008 | 0.1246 |
| Stage | 0.9763 | -0.029738 | 0.9777 | 0.02797 |
| Hist. Grade | 0.928 | 0.090445 | 0.8369 | 0.20585 |
| Residual Tumor | 0.8069 | -0.24452 | 0.8791 | -0.15211 |

**Supplemental Table S3. Predicted therapeutic actions on subtype-specific key driver genes.**

| **Subtype** | **Top Subtype Key Driver Genes** | **Therapeutic Action** |
| --- | --- | --- |
| S-CC | PRMT1, BCL2L12, RUVBL2, BUB1, NOSIP | Inhibition |
| S-immune | CD53, SASH3, BIN2, SPI1, LAIR1 | Activation |
| S-LOC | SHARPIN, HSF1, MAF1, CYHR1, ZC3H3 | Inhibition |
| S-ECM | THBS2, COL5A2, FBN1, COL5A1, COL3A1 | Inhibition |
|  | NCAPH, TEKT1, KIF2C, BUB1, DLGAP5 | Activation |
| S-CC-2 | NCAPH, TOP2A, TPX2, AURKB, RACGAP1 | Inhibition |
|  | CD53, PTPRC, SPI1, SASH3, BIN2 | Activation |
| S-cilium | TEKT1, TUBA4B, DNAI1, RSPH4A, C1orf158 | Activation |
| S-motif | ACSL5, C21orf63, LTC4S, AHDC1, C15orf52 | Inhibition |
|  | ABT1, BAT3, BAT2, ZNF192, ZKSCAN4 | Activation |

**Supplemental Table S4. Number of differentially expressed genes, miRNAs, methylated positions, and amplifications and deletions for each subtype compared to the rest of the samples**

| **Subtype** | **Overexpr Genes** | **Underexpr Genes** | **# DE-miRNAs** | **# DMPs** | **# Amplified Genes** | **# Deleted Genes** |
| --- | --- | --- | --- | --- | --- | --- |
| S-CC | 416 | 230 | 2 | 91 | 541 | 3104 |
| S-immune | 691 | 304 | 10 | 1180 | 1006 | 5020 |
| S-LOC | 332 | 277 | 8 | 241 | 510 | 4515 |
| S-ECM | 1404 | 822 | 98 | 1710 | 461 | 3472 |
| S-CC-2 | 1620 | 1749 | 48 | 3829 | 703 | 4548 |
| S-cilium | 444 | 523 | 11 | 874 | 1237 | 3664 |
| S-motif | 186 | 315 | 26 | 442 | 479 | 5297 |

**Supplemental Table S5. Overlap between subtype DEGs or key drivers and genes significantly correlated (Spearman p < 0.05) with subtype DE-miRNAs**

|  | **DEGs (Up)** | | **DEGs (Down)** | | **Key Drivers (Up)** | | **Key Drivers (Down)** | |
| --- | --- | --- | --- | --- | --- | --- | --- | --- |
| **Subtype** | **% Overlap** | **p** | **% Overlap** | **p** | **% Overlap** | **p** | **% Overlap** | **p** |
| S-CC | 28.37% | 3.54E-151 | 0.43% | 1 | 76.00% | 1.28E-65 | 0% | 1 |
| S-immune | 65.56% | 0 | 14.47% | 9.20E-32 | 98.77% | 1.70E-94 | 34.38% | 1.11E-11 |
| S-LOC | 14.16% | 3.61E-40 | 2.89% | 3.32E-05 | 23.68% | 6.04E-09 | 2.63% | 1 |
| S-ECM | 72.29% | 0 | 46.35% | 3.00E-130 | 95.79% | 5.46E-154 | 81.93% | 7.58E-45 |
| S-CC-2 | 40.01% | 0 | 63.01% | 0 | 64.79% | 2.81E-96 | 86.07% | 4.65E-128 |
| S-cilium | 8.33% | 1.77E-09 | 32.50% | 1.81E-153 | 7.32% | 1 | 73.02% | 4.68E-60 |
| S-motif | 18.28% | 5.10E-14 | 44.44% | 2.16E-117 | 23.53% | 2.07E-01 | 79.59% | 8.01E-46 |

DEG, differentially expressed genes; FE, fold enrichment

**Supplemental Table S6. Overlap between subtype DEGs or key drivers and subtype cis-eDMP genes**

|  | **DEGs (Up)** | | **DEGs (Down)** | | **Key Drivers (Up)** | | **Key Drivers (Down)** | |
| --- | --- | --- | --- | --- | --- | --- | --- | --- |
| **Subtype** | **% Overlap** | **p** | **% Overlap** | **p** | **% Overlap** | **p** | **% Overlap** | **p** |
| S-CC | 5.53% | 1.43E-38 | 1.74% | 3.19E-06 | 18.00% | 1.44E-19 | 7.14% | 7.33E-05 |
| S-immune | 7.09% | 2.42E-57 | 2.30% | 1.91E-07 | 13.58% | 3.09E-16 | 0.00% | 1.00E+00 |
| S-LOC | 0.30% | 1.67E-02 | 2.53% | 2.14E-07 | 0.00% | 1 | 2.63% | 5.60E-02 |
| S-ECM | 3.99% | 1.78E-40 | 3.89% | 1.78E-25 | 5.26% | 5.54E-09 | 0.00% | 1.00E+00 |
| S-CC-2 | 9.38% | 2.14E-118 | 5.95% | 5.23E-96 | 13.62% | 3.51E-26 | 11.44% | 3.99E-26 |
| S-cilium | 2.25% | 1.64E-14 | 19.50% | 2.70E-144 | 0.00% | 1 | 52.38% | 2.61E-61 |
| S-motif | 2.69% | 1.96E-07 | 12.38% | 3.17E-70 | 23.53% | 4.74E-10 | 28.57% | 1.07E-29 |

DEG, differentially expressed genes; FE, fold enrichment

**Supplemental Table S7. Overlap between subtype DEGs or key drivers and predicted amplified or deleted genes**

|  | **DEGs (Up)** | | **DEGs (Down)** | | **Key Drivers (Up)** | | **Key Drivers (Down)** | |
| --- | --- | --- | --- | --- | --- | --- | --- | --- |
| **Subtype** | **% Overlap** | **p** | **% Overlap** | **p** | **% Overlap** | **p** | **% Overlap** | **p** |
| S-CC | 2.40% | 6.17E-02 | 15.22% | 1.23E-04 | 0.00% | 1.00E+00 | 32.14% | 1.94E-04 |
| S-immune | 3.04% | 2.31E-01 | 16.78% | 2.24E-02 | 2.47% | 6.14E-01 | 18.75% | 2.13E-01 |
| S-LOC | 11.14% | 1.71E-23 | 18.77% | 2.13E-04 | 36.84% | 2.12E-17 | 39.47% | 8.29E-06 |
| S-ECM | 1.14% | 5.71E-01 | 15.82% | 3.31E-11 | 0.53% | 8.93E-01 | 22.89% | 8.33E-05 |
| S-CC-2 | 4.51% | 4.53E-13 | 15.72% | 3.33E-08 | 6.57% | 3.14E-05 | 13.93% | 1.64E-01 |
| S-cilium | 7.21% | 1.30E-05 | 28.11% | 1.57E-35 | 2.44% | 7.28E-01 | 38.10% | 6.29E-10 |
| S-motif | 4.84% | 4.73E-04 | 8.89% | 9.95E-01 | 0.00% | 1.00E+00 | 0.00% | 1.00E+00 |

DEG, differentially expressed genes; FE, fold enrichment

**Supplemental Table S8. Presence of amplified and deleted genes commonly associated with HGSOC tumorigenesis for all subtypes.**

| **Amp/Del** | **Subtypes** |
| --- | --- |
| CCNE1 (amp) | S-immune, S-ECM, S-CC-2, S-cilium, S-motif |
| AKT1/2 (amp) | S-LOC, S-cilium |
| MYC (amp) | S-CC, S-LOC, S-CC-2, S-cilium, S-motif |
| BRCA1/2 (del) | S-immune, S-CC-2 |
| NF1 (del) | S-CC, S-immune, S-LOC, S-ECM, S-cilium, S-motif |
| RB1 (del) | S-CC, S-immune, S-LOC, S-CC-2, S-cilium |
| PTEN (del) | S-CC, S-immune, S-LOC, S-cilium, S-motif |

**Supplemental Table S9. Functional annotation enrichment of DEGs between subtype vs. normal ovarian tissue used for drug repositioning analysis.**

| **Subtype** | **DEG Direction** | **DEG Count** | **Enrichment Category** | **Corrected P** | **Fold Enrichment** |
| --- | --- | --- | --- | --- | --- |
| S-CC | Down | 106 | GO: CELL SUBSTRATE JUNCTION | 1.70E-02 | 6.23 |
|  | Up | 155 | GO: MITOTIC CELL CYCLE | 4.50E-26 | 8.22 |
|  | Up | 155 | GO: CELL CYCLE PROCESS | 2.90E-24 | 6.43 |
| S-immune | Down | 115 | GO: ORGAN MORPHOGENESIS | 6.40E-04 | 4.49 |
|  | Down | 115 | GO: TISSUE MORPHOGENESIS | 4.30E-03 | 5.40 |
|  | Up | 157 | GO: IMMUNE RESPONSE | 3.20E-44 | 8.25 |
|  | Up | 157 | GO: IMMUNE SYSTEM PROCESS | 1.30E-39 | 5.39 |
| S-LOC | Down | 323 | GO: CILIUM MORPHOGENESIS | 5.80E-02 | 4.85 |
|  | Up | 283 | GO: MITOTIC CELL CYCLE | 3.60E-08 | 3.79 |
|  | Up | 283 | GO: CELLULAR RESPONSE TO DNA DAMAGE STIMULUS | 1.10E-07 | 3.83 |
| S-ECM | Down | 273 | GO: DNA METABOLIC PROCESS | 1.70E-08 | 3.97 |
|  | Down | 273 | GO: CHROMOSOME ORGANIZATION | 4.80E-07 | 3.29 |
|  | Up | 464 | GO: EXTRACELLULAR MATRIX | 7.30E-58 | 9.29 |
|  | Up | 464 | GO: PROTEINACEOUS EXTRACELLULAR MATRIX | 3.20E-53 | 9.91 |
| S-CC-2 | Down | 381 | GO: IMMUNE RESPONSE | 4.90E-59 | 6.03 |
|  | Down | 381 | GO: IMMUNE SYSTEM PROCESS | 8.10E-50 | 4.02 |
|  | Up | 266 | GO: CHROMOSOME ORGANIZATION | 4.50E-11 | 3.79 |
|  | Up | 266 | GO: MICROTUBULE CYTOSKELETON | 3.20E-08 | 3.36 |
|  | Up | 266 | GO: MITOTIC CELL CYCLE | 3.40E-07 | 3.77 |
| S-cilium | Down | 148 | Reactome: GENERIC TRANSCRIPTION PATHWAY | 1.90E-15 | 10.81 |
|  | Down | 148 | GO: NUCLEIC ACID BINDING TRANSCRIPTION FACTOR ACTIVITY | 4.70E-04 | 3.41 |
|  | Up | 29 | GO: NAD BIOSYNTHETIC PROCESS | 1.00E+00 | 104.93 |
|  | Up | 29 | GO: ORGANONITROGEN COMPOUND BIOSYNTHETIC PROCESS | 1.00E+00 | 5.38 |
| S-motif | Down | 321 | Reactome: CELL CYCLE MITOTIC | 1.90E-07 | 5.30 |
|  | Down | 321 | Reactome: DNA REPLICATION | 3.30E-07 | 6.98 |
|  | Up | 146 | CAGGTG E12 Q6 | 2.10E-01 | 2.17 |
|  | Up | 146 | ARGGGTTAA UNKNOWN | 2.40E-01 | 9.39 |

**Supplemental Table S10. Top 5 repositioned drugs for each subtype.**

| **Subtype** | **Rank** | **Drug Name** |
| --- | --- | --- |
| S-CC | 1 | BRD-K26664453 |
|  | 2 | tyrphostin ag 1478 |
|  | 3 | brd-k73008154 |
|  | 4 | bms-754807 |
|  | 5 | curcubitacin-i |
|  | 6 | ncgc00185684-02 |
|  | 7 | dg-041 |
|  | 8 | phorbol-12-myristate-13-acetate |
|  | 9 | phorbol-12-myristate-13-acetate (pma) |
|  | 10 | brd-k44242139 |
| S-immune | 1 | BRD-K02847640 |
|  | 2 | BRD-K38188442 |
|  | 3 | BRD-K15240833 |
|  | 4 | BRD-K40455893 |
|  | 5 | BRD-K21630495 |
|  | 6 | BRD-K11401851 |
|  | 7 | BRD-K88334823 |
|  | 8 | BRD-K95526045 |
|  | 9 | BRD-K28162668 |
|  | 10 | barasertib |
| S-LOC | 1 | brd-k44242139 |
|  | 2 | hy-10044 |
|  | 3 | brd-k44732214 |
|  | 4 | BRD-K74710236 |
|  | 5 | azd8055 |
|  | 6 | chr 2797 |
|  | 7 | BRD-K44432556 |
|  | 8 | BRD-K57080016 |
|  | 9 | a-1065 |
|  | 10 | BRD-K12184916 |
| S-ECM | 1 | BRD-K33130522 |
|  | 2 | BRD-K95063618 |
|  | 3 | BRD-K52341396 |
|  | 4 | BRD-K94064112 |
|  | 5 | BRD-K48005755 |
|  | 6 | BRD-K94300226 |
|  | 7 | ropinirole |
|  | 8 | BRD-K68201904 |
|  | 9 | BRD-K02847640 |
|  | 10 | BRD-K19362120 |
| S-CC-2 | 1 | t542500 |
|  | 2 | kuc104495 kuc104495n |
|  | 3 | camptothecin |
|  | 4 | kuc104135 kuc104135n |
|  | 5 | BRD-K06666320 |
|  | 6 | hy-50940 |
|  | 7 | \n-((1h-naphtho[2,3-d]imidazol-2-yl)methyl)-2-morpholino-9-(thiophen-3-yl)-9h-purin-6-amine\ |
|  | 8 | 2-morpholino-n-((5-nitro-1h-benzo[d]imidazol-2-yl)methyl)-9-(thiophen-3-yl)-9h-purin-6-amine |
|  | 9 | BRD-A30437061 |
|  | 10 | n-((5-chloro-1h-benzo[d]imidazol-2-yl)methyl)-2-morpholino-9-(thiophen-3-yl)-9h-purin-6-amine |
| S-cilium | 1 | BRD-K43298786 |
|  | 2 | BRD-K81146569 |
|  | 3 | BRD-K46774516 |
|  | 4 | BRD-K67769696 |
|  | 5 | BRD-K69611470 |
|  | 6 | BRD-K74639017 |
|  | 7 | BRD-K49906853 |
|  | 8 | BRD-K59893315 |
|  | 9 | BRD-K44953633 |
|  | 10 | BRD-K33236383 |
| S-motif | 1 | BRD-K76896099 |
|  | 2 | BRD-K80076202 |
|  | 3 | BRD-K82048228 |
|  | 4 | BRD-K15262863 |
|  | 5 | BRD-K62561943 |
|  | 6 | prilocaine |
|  | 7 | BRD-K07933458 |
|  | 8 | BRD-K57443339 |
|  | 9 | BRD-K68370959 |
|  | 10 | BRD-K29054201 |
